# Rapid Surface Reconstruction of In_2_S_3_ Photoanode via Flame Treatment for Enhanced Photoelectrochemical Performance

**DOI:** 10.1002/adma.202403164

**Published:** 2024-05-30

**Authors:** Yoo Jae Jeong, Runfa Tan, Seongsik Nam, Jong Ho Lee, Sung Kyu Kim, Tae Gyu Lee, Seong Sik Shin, Xiaolin Zheng, In Sun Cho

**Affiliations:** ^1^ Department of Energy Systems Research Ajou University Suwon 16499 Republic of Korea; ^2^ Department of Material Science & Engineering Ajou University Suwon 16499 Republic of Korea; ^3^ Department of Nano Engineering Department of Nano Science and Technology SKKU Advanced Institute of Nanotechnology (SAINT) Sungkyunkwan University Suwon 16419 Republic of Korea; ^4^ SKKU Institute of Energy Science and Technology (SIEST) Sungkyunkwan University Suwon 16419 Republic of Korea; ^5^ Department of Nanotechnology and Advanced Materials Engineering Sejong University Seoul 05006 Republic of Korea; ^6^ Department of Mechanical Engineering Stanford University Stanford CA 94305 USA

**Keywords:** flame treatment, In_2_S_3_ photoanode, photoelectrochemical performance, stability, surface reconstruction

## Abstract

Surface reconstruction, reorganizing the surface atoms or structure, is a promising strategy to manipulate materials' electrical, electrochemical, and surface catalytic properties. Herein, a rapid surface reconstruction of indium sulfide (In_2_S_3_) is demonstrated via a high‐temperature flame treatment to improve its charge collection properties. The flame process selectively transforms the In_2_S_3_ surface into a diffusionless In_2_O_3_ layer with high crystallinity. Additionally, it controllably generates bulk sulfur vacancies within a few seconds, leading to surface‐reconstructed In_2_S_3_ (sr‐In_2_S_3_). When using those sr‐In_2_S_3_ as photoanode for photoelectrochemical water splitting devices, these dual functions of surface In_2_O_3_/bulk In_2_S_3_ reduce the charge recombination in the surface and bulk region, thus improving photocurrent density and stability. With optimized surface reconstruction, the sr‐In_2_S_3_ photoanode demonstrates a significant photocurrent density of 8.5 mA cm^−2^ at 1.23 V versus a reversible hydrogen electrode (RHE), marking a 2.5‐fold increase compared to pristine In_2_S_3_ (3.5 mA cm^−2^). More importantly, the sr‐In_2_S_3_ photoanode exhibits an impressive photocurrent density of 7.3 mA cm^−2^ at 0.6 V versus RHE for iodide oxidation reaction. A practical and scalable surface reconstruction is also showcased via flame treatment. This work provides new insights for surface reconstruction engineering in sulfide‐based semiconductors, making a breakthrough in developing efficient solar‐fuel energy devices.

## Graphical abstract

A rapid surface reconstruction strategy is proposed to enhance the charge collection efficiency and stability of indium sulfide (In_2_S_3_) photoanode via a high‐temperature flame treatment. This method selectively transforms the In_2_S_3_ surface into a diffusionless In_2_O_3_ and generates bulk sulfur vacancies, resulting in surface‐reconstructed In_2_S_3_ (sr‐In_2_S_3_). The sr‐In_2_S_3_ exhibits an impressive photocurrent density of 8.5 mA cm^−2^, outperforming previously reported sulfide‐based photoanodes.

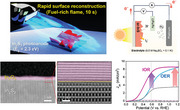

## 1 Introduction

Photoelectrochemical (PEC) water splitting utilizes solar energy to convert water into green hydrogen (H_2_) in one system.^[^ ^1^, ^2^ ^]^ Realization of PEC devices requires cost‐effective photoelectrode materials with high solar‐to‐hydrogen conversion efficiency and stable performance. One of the primary challenges in PEC water splitting is the sluggish oxygen evolution reaction (OER) on the photoanode, which is attributed to its slow 4‐electron transfer reaction compared to the 2‐electron transfer of hydrogen evolution reaction.^[^ ^3^, ^4^ ^]^ Additionally, this leads to the production of economically less valuable O_2_. Recently, to overcome these bottlenecks in OER, alternative photooxidation reactions such as urea oxidation, methanol oxidation, and iodide oxidation reaction (IOR) have been explored due to their favorable reaction kinetics and the generation of economically valuable products.^[^ ^5^, ^6^, ^7^ ^]^ In particular, IOR is a promising oxidation reaction due to its lower thermodynamic potential of 0.54 V and rapid 2‐electron transfer reaction. Moreover, the resulting liquid product, triiodide (I_3_^−^), holds high value in diverse energy industries, such as pharmaceuticals, disinfectants, and catalysts, and is relatively stable and convenient to handle compared to gas products.^[^ ^8^, ^9^ ^]^

Various materials, including metal oxides, sulfides, and nitrides, have been investigated to develop an efficient photoanode.^[^ ^10^, ^11^, ^12^, ^13^ ^]^ Among them, indium sulfide (In_2_S_3_) has recently gained attention because of its excellent light absorption coefficient (≈10^4^ cm^−1^), high electrical conductivity, and outstanding photoelectric conversion efficiency.^[^ ^14^, ^15^ ^]^ However, the In_2_S_3_ photoanode still suffers from excessive charge carrier recombination losses and deficient catalytic active sites, limiting its PEC performance. Numerous efforts have been attempted to address these limitations on the In_2_S_3_ photoanode, including intrinsic/extrinsic doping,^[^ ^16^ ^]^ forming type‐II heterojunction,^[^ ^17^, ^18^ ^]^ and defect engineering.^[^ ^19^ ^]^ Nevertheless, the current PEC performance of the In_2_S_3_ photoanode is still unsatisfactory, especially the photocurrent and stability.

Surface reconstruction (i.e., tailoring surface structure and composition) is an effective strategy to enhance the electrical, electrochemical, optical, and catalytic properties because the near‐surface region of the photoelectrode is in direct contact with the electrolyte and gets involved in the chemical redox reaction.^[^ ^20^ ^]^ For example, Meng et al. reported a facile heat treatment of Zn_10_In_16_S_34_ photoanode that induces surface oxygen doping and metal vacancy to boost charge transport and transfer efficiencies.^[^ ^21^ ^]^ The same group utilized an ultrasonication‐assisted method to reconstruct the surface of the CdIn_2_S_4_ photoanode by utilizing Zn‐modified Cd defects. This approach facilitated charge transfer and increased the number of surface‐active sites, achieving a photocurrent density of 5.30 mA cm^−2^.^[^ ^22^ ^]^ Cao et al. reported that In_2_S_3_/In_2_O_3_ photoanode with a 3D porous surface demonstrated significantly improved photocurrent density by combining heterostructure design with increased surface area.^[^ ^23^ ^]^ A nonstoichiometric In_2_S_3_ surface was formed in situ through a straightforward solvothermal annealing process, leading to a type‐II heterojunction with a high‐quality interface. This reconstructed surface effectively enhances surface catalytic kinetics and facilitates charge separation.^[^ ^24^ ^]^ All these previously reported methods demonstrated that the surface is efficacious in improving electrochemical catalytic performance. However, they generally require longer treatment times at low temperatures to protect the samples and substrates against thermal damage and phase transformation. In addition, such methods frequently require a vacuum or inert gas atmosphere environment and involve complex multistep processes. Therefore, developing an effective and rapid process for surface reconstruction under ambient conditions is desirable to enhance the PEC performance.

In this study, inspired by the susceptibility of sulfide materials to oxidation, we demonstrate a rapid surface reconstruction of the In_2_S_3_ photoanode via a high‐temperature flame treatment (≈1,100 °C, 10 s) under ambient conditions to maximize its charge collection properties. The fuel‐rich flame with an ultrafast heating rate enables dual functions: 1) it converts the In_2_S_3_ surface into an active and stable In_2_O_3_ layer (≈5 nm), and 2) it generates sulfur vacancies at the bulk region without damaging deteriorating other properties. These nanoscale changes alter the surface and electronic structures of the In_2_S_3_ photoanode, enhancing charge collection properties and even photocurrent stability. Notably, the resulting surface‐reconstructed In_2_S_3_ (sr‐In_2_S_3_) exhibits significantly improved PEC performance, achieving a photocurrent density of 8.5 mA cm^−2^ at 1.23 V versus reversible hydrogen electrode (RHE) for OER and 7.3 mA cm^−2^ at 0.6 V versus RHE for IOR under simulated sunlight illumination (100 mW cm^−2^). Finally, we showcase a practical and scalable surface reconstruction via flame treatment for IOR, demonstrating the viability of the flame‐based surface reconstruction method for developing high‐performance photoelectrodes.

## 2 Results and Discussions

### 2.1 Surface Reconstruction of In_2_S_3_ Photoanode via a Rapid Flame Treatment

**Figure 1:**
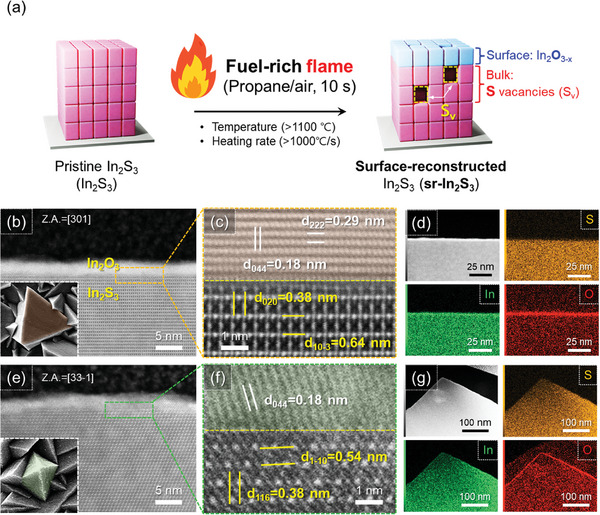
Flame‐based surface reconstruction and its impact on the morphology and surface structure. a) Schematic process of the surface reconstruction via a flame treatment. High‐resolution transmission electron microscopy (HR‐TEM) and energy dispersive spectroscopy (EDS) mapping of b–d) square‐ and e–g) pyramid‐shaped grains.

**Figure** **1**a shows the schematic surface‐reconstruction process of the In_2_S_3_ photoanode via a rapid flame treatment under ambient conditions. The In_2_S_3_ polycrystalline photoanode (≈4.5 µm thick) was grown on the fluorine‐doped tin oxide (FTO) substrate by hydrothermal method. The detailed growth and optimization process are shown in Figures S1 and S2 (Supporting Information). Next, the In_2_S_3_ photoanode was treated with a fuel‐rich flame (≈1100 °C) for 10 s to induce the surface reconstruction, i.e., which transforms the top In_2_S_3_ layer into a conformal layer of In_2_O_3−_ *_x_* and generates sulfur (S) vacancies simultaneously at the bulk region (Figure S3, Supporting Information). The effect of flame treatment on the morphology of In_2_S_3_ photoanode is illustrated by the scanning electron microscopy (SEM) images in Figure S4 (Supporting Information). Before the flame treatment, the hydrothermal grown In_2_S_3_ had both square‐ and pyramid‐shaped grains, and there was no apparent morphology change for the first 10 s of flame treatment. The flame‐treated In_2_S_3_ photoanode maintains a homogeneous interface between grains, intimate contact with the FTO substrate, and exhibits good crystallinity (Figures S5–S7, Supporting Information). Closer inspection by high‐resolution transmission electron microscopy (TEM) shows a ≈5 nm thick crystalline and homogeneous In_2_O_3_ layer formed on the surface of the In_2_S_3_ (Figure 1b,c,e,f). Notably, the whole layer of the In_2_O_3_, even at the edge parts, exhibits clear lattice fringes, indicating a highly crystalline nature of the surface‐reconstructed layer (Figure S8, Supporting Information). The formed In_2_O_3_ layer has a heteroepitaxial relationship with the bottom In_2_S_3_, as shown by the lattice images at the interface (Figure S9, Supporting Information), which may be attributed to the ultrafast heating rate (>1000 °C s^−1^) and extremely high temperature (>1100 °C) of the flame.^[^ ^25^ ^]^ The lattice spacings of 0.18 and 0.29 nm observed for the top layer in Figure 1c,f correspond to the (044) and (222) planes of the cubic In_2_O_3_. For the bottom part of the interfaces, the lattice spacings of 0.38 and 0.64 nm correspond to the (020) and (10–3) of tetragonal In_2_S_3_ in square‐shaped grains, while the lattice spacings of 0.54 and 0.38 nm correspond to the (1–10) and (116) in pyramid‐shaped grains, respectively. Additionally, the energy dispersive spectroscopy (EDS) mapping results confirm the homogeneous and uniform formation of the In_2_O_3_ layer with a distinct interface at the entire In_2_S_3_ surface (Figure 1d–g and Figure S10 (Supporting Information)).

### 2.2 Crystalline Structure, Surface, and Defect Analyses

**Figure 2:**
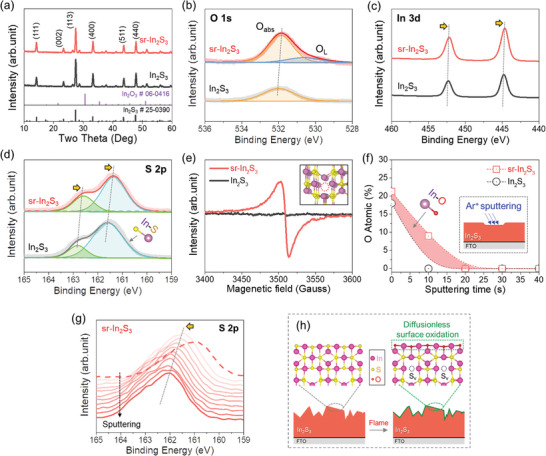
Crystalline structure, surface, and defect analyses of sr‐In_2_S_3_ photoanode. a) XRD patterns with JCPDS card number of In_2_S_3_ and In_2_O_3_. XPS spectra of b) O 1s, c) In 3d, and d) S 2p. e) Electron paramagnetic resonance (EPR) spectra. f) O atomic and g) S 2p spectra of sr‐In_2_S_3_ (10 s) from XPS depth profiles. h) Schematic illustration of surface reconstruction on sr‐In_2_S_3_ photoanode.

The X‐ray diffraction (XRD) and X‐ray photoelectron spectroscopy (XPS) analyses were performed to investigate the crystal structure, surface chemical states, and chemical bonding nature of the sr‐In_2_S_3_. As shown in **Figure** **2**a, the XRD pattern of 0 s (untreated In_2_S_3_) shows phase‐pure tetragonal In_2_S_3_ (JCPDS card no. 25‐0390). The flame‐treated sample, i.e., the sr‐In_2_S_3_ (10 s treatment), exhibited no change in the XRD peaks and positions. Similarly, the Raman spectra also exhibited no difference (Figure S11, Supporting Information). These results indicate that the short flame treatment within 10 s can effectively induce surface reconstruction without damaging the bulk region.

Possible doping or deposition of carbon species on the surface of In_2_S_3_ was checked by XPS C 1s spectra (Figure S12, Supporting Information). The result indicates that there is no incorporation of carbon species. Next, the XPS O 1s spectra (Figure 2b) were deconvoluted into two peaks at 530.2 and 532.0 eV, corresponding to lattice oxygen (O_L_) and adsorbed O_2_ (O_ads_), respectively.^[^ ^26^, ^27^ ^]^ The O_L_ and O_ads_ peaks increased with flame treatment (10 s), indicating surface oxidation. Additionally, as shown in Figure 2c and Figure S13 (Supporting Information), the In 3d peaks negatively shifted and increased in peak intensity with the flame treatment, indicating the removal of surface OH groups and progressive oxidation (i.e., the formation of In─O─In bonding).^[^ ^21^, ^23^, ^28^, ^29^ ^]^ Furthermore, two sulfate groups (SO_4_^2−^) peaks at 168.5 and 169.8 eV were observed for flame‐treated samples, which is also attributed to the surface oxidation (Figure S14, Supporting Information). Notably, this nonmetallic anionic group is believed to influence the electronic structure of the active site, thus reducing the overpotential for the OER.^[^ ^30^, ^31^ ^]^

On the other hand, the two peaks at 161.6 and 162.8 eV in the S 2p spectra (i.e., the valence state of S^2−^, Figure 2d and Figure S13 (Supporting Information)) exhibited a negative shift and intensity decrease with the flame treatment, which is attributed to the formation of S vacancies and the loss of S elements.^[^ ^32^, ^33^ ^]^ The presence of S vacancies was further confirmed by electron paramagnetic resonance (EPR) spectroscopy (Figure 2e). The sr‐In_2_S_3_ (10 s) exhibited a greatly enhanced EPR signal than the 0 s, evidencing the existence of S vacancies.^[^ ^34^, ^35^ ^]^ The slightly asymmetric EPR spectrum can be attributed to the other type of paramagnetic substance coexisting with S vacancies (possibly O vacancies in In_2_O_3_ phase) and the defect‐induced interaction between the electron spins and local environment changes.^[^ ^23^, ^36^, ^37^, ^38^ ^]^ The XPS depth profiles were further investigated (Figure 2f and Figure S15 (Supporting Information)). After flame treatment, the sr‐In_2_S_3_ (10 s) exhibits a slightly deeper depth of O atoms than the pristine In_2_S_3_ photoanode (0 s), demonstrating the presence of a diffusionless In_2_O_3_ layer on the outer surface. Interestingly, sr‐In_2_S_3_ photoanode (10 s) showed a gradual S 2p peak shift toward the high binding energy with the increase of the Ar sputtering (i.e., along the depth) (Figure 2g and Figure S16 (Supporting Information)). This result suggests that the S vacancies have a gradient distribution through the bulk region.^[^ ^21^, ^39^ ^]^ As a result, these XPS and the above TEM analyses reveal that a short flame duration (10 s) favors the formation of the In_2_O_3_ layer and S vacancies at the bulk region. Notably, the S vacancies play a significant role in the physicochemical properties of the material for (photo)electrochemistry.^[^ ^40^, ^41^ ^]^ In particular, the S vacancies increase the charge carrier density and electrical conductivity, enhancing charge transport properties for PEC devices.^[^ ^42^, ^43^ ^]^

Figure 2h shows a surface reconstruction mechanism via diffusionless surface oxidation. The sulfur ions near the surface region rapidly evaporate, and oxygen atoms from the flame ionize and incorporate into the surface structure, thus generating S vacancies and forming a crystallized In_2_O_3_ layer. Even though the temperature is sufficient for rapid diffusion toward the bulk region, the O^2−^ diffusion is critically limited due to the short duration time, rapid cooling, and low O_2_ concentration in the flame (fuel‐rich condition). Therefore, only a few nanometers thick In_2_O_3_ layers form at the surface region (Figures S17 and S18, Supporting Information).

### 2.3 PEC Performance

**Figure 3:**
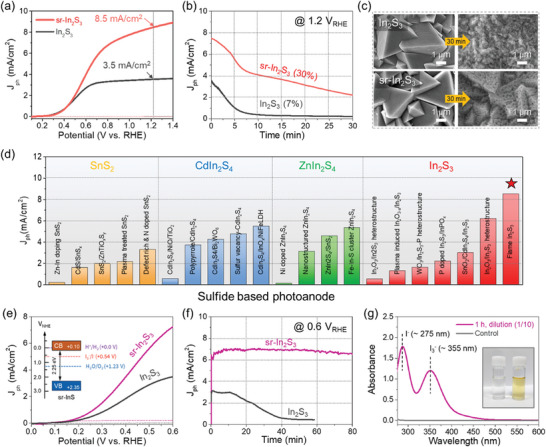
PEC performance of sr‐In_2_S_3_ photoanode. a) Photocurrent density–potential (*J*–*V*) curves measured in 0.5 m Na_2_SO_4_ under AM 1.5G (100 mW cm^−2^) illumination. b) Photostability test using 0.5 m Na_2_SO_4_. c) SEM images of In_2_S_3_ and sr‐In_2_S_3_ before/after the PEC stability test (30 min). d) Comparison of photocurrent density of sulfide‐based photoanode reported in recent year. e) *J*–*V* curves measured in 0.5 m Na_2_SO_4_ with 0.1 m KI. f) Photostability stability test of sr‐In_2_S_3_ and In_2_S_3_ under 0.6 V versus RHE. g) UV–vis absorbance spectra of the electrolyte with the corresponding electrolyte photograph: Control (0.5 m Na_2_SO_4_ + 0.1 m KI) and electrolyte after 1 h PEC operation. The testing conditions in (d) were summarized in Table S1 (Supporting Information).

The effect of surface reconstruction on PEC performance is evaluated by measuring photocurrent density–potential (*J*–*V*) curves in a three‐electrode setup with 0.5 m Na_2_SO_4_ (pH 6.8) as the electrolyte (without scavenger) under AM 1.5G illumination. As shown in **Figures** **3**a and S19 (Supporting Information), the photocurrent density (*J*_ph_) of sr‐In_2_S_3_ (10 s) photoanode showed a significantly improved value of 8.5 mA cm^−2^ compared to the In_2_S_3_ (3.5 mA cm^−2^) at 1.23 V versus RHE. There was no difference in the onset potential of In_2_S_3_ and sr‐In_2_S_3_. Photocurrent stability is a crucial requirement for achieving viable PEC applications. All sulfide semiconductors generally suffer from severe degradation or photocorrosion due to the self‐oxidation process (S^2−^ + 2h^+^ = S), deteriorating the PEC stability (Figures S20 and S21, Supporting Information).^[^ ^44^ ^]^ In this regard, we investigated the photocorrosion behavior and photostability of the In_2_S_3_ and sr‐In_2_S_3_ photoanodes by measuring the photocurrent–time (*J*–*t*) curve (Figure 3b and Figure S22 (Supporting Information)). Interestingly, the reconstructed surface in the sr‐In_2_S_3_ photoanode suppresses the degradation rate relatively compared to the In_2_S_3_ counterpart, i.e., the sr‐In_2_S_3_ maintained ≈30% of the initial photocurrent value even after 30 min (4 times higher than the In_2_S_3_, 7%). The SEM images (after 30 min PEC operation) show that the In_2_S_3_ photoanode exhibited damaged and collapsed morphology entirely (Figure 3c). By contrast, the sr‐In_2_S_3_ photoanode retained its original morphology despite many pores. These results indicate improved photoelectrochemical stability of sr‐In_2_S_3_ against photocorrosion. Therefore, surface reconstruction of In_2_S_3_ via flame treatment effectively suppresses photocorrosion. Here, it should be noted that there is still room for enhancing photocurrent stability via an additional surface modification, such as the deposition of a protective layer and electrocatalysts.^[^ ^45^, ^46^, ^47^ ^]^

As shown in Figure 3d and Table S1 (Supporting Information), the *J*_ph_ of the sr‐In_2_S_3_ photoanode was compared to those of sulfide‐based photoanodes reported in recent years (detailed information on the measurement conditions is summarized in Table S1 in the Supporting Information). Notably, photocurrent density values of our sr‐In_2_S_3_ photoanode (8.5 mA cm^−2^) are the highest values among all reported sulfide‐based photoanodes, including SnS_2_, In_2_S_3_, CdIn_2_S_4_, and ZnIn_2_S_4_.^[^ ^19^, ^26^, ^27^, ^39^, ^48^, ^49^, ^50^, ^51^, ^52^, ^53^, ^54^, ^55^, ^56^, ^57^, ^58^, ^59^, ^60^, ^61^, ^62^ ^]^ Therefore, this result demonstrated the effectiveness of the surface reconstruction via flame treatment to improve the PEC performance of the In_2_S_3_ photoanode.

We tested the IOR activity using the sr‐In_2_S_3_ photoanode in the 0.5 m Na_2_SO_4_ + 0.1 m potassium iodide (KI) electrolyte. Electrochemical activity in the dark condition confirms that the IOR is kinetically more favorable than OER (Figure S23, Supporting Information). Significantly, as shown in Figure 3e and Figure S24 (Supporting Information), the sr‐In_2_S_3_ photoanode demonstrated significantly improved PEC IOR performance of 7.3 mA cm^−2^ at 0.6 V versus RHE compared to the In_2_S_3_ of 3.5 mA cm^−2^, accompanied by 200 mV cathodic shift in the onset potential due to the fast IOR kinetics. Furthermore, the sr‐In_2_S_3_ photoanode exhibited much higher applied bias photon‐to‐current efficiency (ABPE: 4.4%) and transfer efficiency (86%) for IOR than those for OER (ABPE: 2.5% and transfer efficiency: 44%) at 0.6 V versus RHE (Figure S25, Supporting Information). This result indicates the superior PEC IOR performance of the sr‐In_2_S_3_ photoanode, even comparable to the state‐of‐the‐art performances.^[^ ^9^, ^63^, ^64^ ^]^ Therefore, the superior PEC IOR performance of the sr‐In_2_S_3_ underscores the advantages of our flame treatment method. The photocurrent stability of PEC IOR was also checked (Figure 3f). Impressively, the sr‐In_2_S_3_ photoanode exhibited a much more stable photocurrent density with little degradation during 80 min of operation. Compared to the PEC OER, the sr‐In_2_S_3_ photoanode showed greatly improved stability, demonstrating the effectiveness of the sr‐In_2_S_3_ photoanode for PEC IOR (Figure S26, Supporting Information). UV–vis absorbance spectra of collected electrolyte were examined to clarify the production of I_3_^−^ during PEC IOR operation, and inset images show the corresponding electrolyte photograph (Figure 3g). The peaks at ≈275, 355, and 450 nm in I_2_ solution (i.e., reference solution) correspond to I^−^, I_3_^−^, and I_2_, respectively (Figure S27, Supporting Information).^[^ ^65^ ^]^ After 1 h of illumination, the production of I_3_^−^ was observed by the increase in peak intensity at 335 nm (with a tenfold dilute solution), verifying the PEC IOR of the sr‐In_2_S_3_ photoanode. Figures S28–S30 (Supporting Information) show gas evolution and the Faradaic efficiency (FE) of sr‐In_2_S_3_ photoanode via IOR. The FEs of H_2_ and I_3_^−^ on sr‐In_2_S_3_ photoanode exhibited ≈63% and 55% (nearly 1:1 ratio), indicating hydrogen and triiodide evolution through IOR. The slightly low FE of the sr‐In_2_S_3_ photoanode is attributed to the triiodide back reaction into iodide at the Pt counter electrode.^[^ ^9^, ^64^ ^]^

### 2.4 Optical Properties and Electrochemical Characterizations

**Figure 4:**
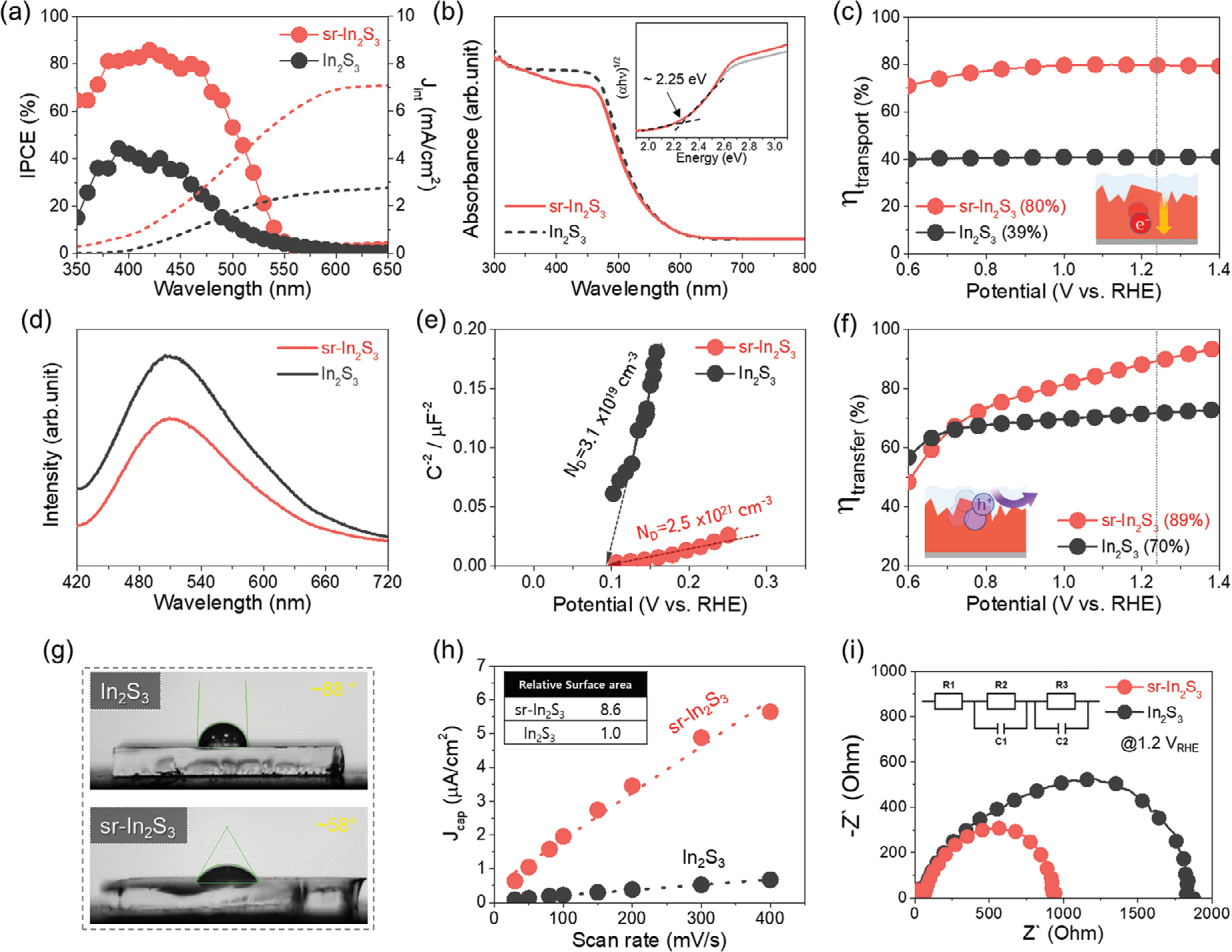
Optical properties and electrochemical characterizations. a) Incident photoelectron conversion efficiency (IPCE) curves. b) Absorbance spectra and Tauc plot. c) Charge transport efficiency. d) Photoluminescence (PL) spectra. e) Mott–Schottky analysis. f) Charge transfer efficiency. g) Contact angle analysis using 0.5 m Na_2_SO_4_ + 0.1 m KI electrolyte. h) Electrochemical active surface area (EASA) analysis. i) Potentiostatic electrochemical impedance spectroscopy (PEIS) analysis and the corresponding equivalent circuit model.

The incident photon‐to‐current conversion efficiencies (IPCEs) of In_2_S_3_ and sr‐In_2_S_3_ photoanodes were evaluated (**Figure** **4**a). The IPCE value of sr‐In_2_S_3_ photoanode is much higher than that of In_2_S_3_ photoanode in the whole wavelength range, which is consistent with the result of the *J*–*V* curves. There was no critical difference in IPCE edge wavelengths, indicating that the surface reconstruction has little impact on the bandgap and band edge positions. In addition, the absorbed photon‐to‐current efficiency (APCE) value of sr‐In_2_S_3_ photoanode is higher than that of In_2_S_3_ photoanode, indicating the enhanced charge separation due to surface reconstruction (Figure S31, Supporting Information).

To comprehensively grasp the factors influencing IPCE, the optical and charge collection properties of both In_2_S_3_ and sr‐In_2_S_3_ photoanode were investigated. First, the absorbance spectra (Figure 4b) show that both In_2_S_3_ and sr‐In_2_S_3_ photoanodes exhibit comparable light absorption and bandgap, indicating similar charge generation efficiency. Next, the charge collection (transport and transfer) efficiencies in Figures 4c,f were obtained using 0.5 m Na_2_SO_4_ and Na_2_SO_3_ based on the hole‐scavenger method.^[^ ^66^, ^67^ ^]^ The detailed calculation method can be found in the Experimental Section of the Supporting Information. Significantly, the sr‐In_2_S_3_ photoanode exhibited a superior photocurrent density of 9.5 mA cm^−2^ in the presence of 0.5 m Na_2_SO_3_ (a hole scavenger, Figure S32, Supporting Information). Besides, the onset potential considerably shifted (≈600 mV) to −0.2 V versus RHE in the presence of the hole scavenger. Figure 4c shows that the charge transport efficiency of sr‐In_2_S_3_ (80%) is twice that of In_2_S_3_ (39%). From the photoluminescence (PL) and time‐resolved PL measurements, we found that the sr‐In_2_S_3_ has a lower PL peak intensity and a longer carrier lifetime (Figure 4d and Figure S33 (Supporting Information)). This result indicates that the surface reconstruction suppresses charge recombination, which results from the homogeneous and uniform interfacial S─O bond between the In_2_O_3_ layer and underlying In_2_S_3_.^[^ ^68^ ^]^ The Mott–Schottky analysis shows a positive slope of In_2_S_3_ and sr‐In_2_S_3_ photoanodes, indicating n‐type semiconductor behavior (Figure 4e and Figure S34 (Supporting Information)). More importantly, the sr‐In_2_S_3_ photoanode exhibited a much smaller slope than the In_2_S_3_ counterpart, confirming greatly enhanced charge carrier density (*N*_D_), which is attributed to the generation of S vacancies. Xu et al. reported that bulk S vacancies increase the donor density, thus significantly improving the charge separation (transport) efficiency and OER activity.^[^ ^31^ ^]^ Therefore, the 2 times higher charge transport efficiency of the sr‐In_2_S_3_ photoanode is ascribed to the reduced charge recombination and improved *N*_D_.

Figure 4f shows the charge transfer efficiencies of In_2_S_3_ and sr‐In_2_S_3_ photoanodes as a function of applied potential. The sr‐In_2_S_3_ photoanode exhibits a higher charge transfer efficiency of 89% than the In_2_S_3_ photoanode (70%) at 1.23 V versus RHE. Moreover, under dark conditions, the sr‐In_2_S_3_ photoanode exhibited a much lower OER overpotential and higher activity than the In_2_S_3_ photoanode (Figure S35, Supporting Information). The surface hydrophilicity of sr‐In_2_S_3_ was assessed using the contact angle measurements (Figure 4g). The contact angle of sr‐In_2_S_3_ (≈58%) is lower than that of In_2_S_3_ (≈88%), indicating a more hydrophilic surface of sr‐In_2_S_3_. Electrochemical active surface area (EASA) is further measured to compare the surface‐active sites (Figure 4h and Figure S36 (Supporting Information)). Interestingly, the EASA of sr‐In_2_S_3_ was 8 times larger than that of the In_2_S_3_, signifying an increased number of active sites due to the surface In_2_O_3_ layer. This outcome suggests that the surface In_2_O_3_ layer has point defects like oxygen vacancy, providing a rich source of surface adsorption sites.^[^ ^39^, ^55^ ^]^

Next, potentiostatic electrochemical impedance spectroscopy (PEIS) studies were conducted to confirm the enhanced hole transfer kinetics and charge transport by the reconstructed surface in the sr‐In_2_S_3_ photoanode (Figure 4i and Figures S37 and S38 (Supporting Information)). *R*_trapping_ values of the sr‐In_2_S_3_ photoanode are lower than that of In_2_S_3_ counterpart, indicating the decreased charge recombination of electron/hole pairs. Additionally, the sr‐In_2_S_3_ photoanode exhibited enhanced *C*_bulk_ values at all potential ranges, indicating the increased charge carrier density owing to the S vacancies induced by the flame treatment. Meanwhile, compared to the In_2_S_3_ photoanode, decreased *R*_ct_ values and enhanced *C*_trap_ values on the sr‐In_2_S_3_ photoanode demonstrate the enhanced hole injection kinetics and diminished hole accumulation at the photoanode and electrolyte interface.^[^ ^50^ ^]^ Therefore, these results indicate that both the reconstructed In_2_O_3_ layer and S vacancies via flame treatment could effectively suppress the bulk/surface charge recombination, promoting the charge collection properties and, eventually, the PEC energy conversion efficiency.

### 2.5 Large‐Scale Application of Flame Treatment

**Figure 5:**
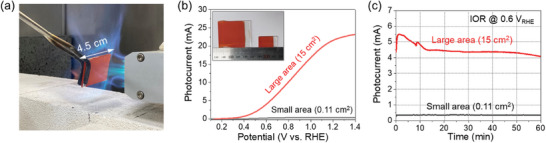
Large‐scale application of flame treatment. a) Photograph of flame treatment on large area photoanode (15 cm^2^). b) Photocurrent of large and small area In_2_S_3_ photoanodes. c) IOR photocurrent stability of the large area sr‐In_2_S_3_ photoanode under 0.6 V versus RHE.

In practical PEC applications, it is essential to fabricate large‐area photoelectrode that exhibits high performance.^[^ ^69^, ^70^ ^]^ In this context, we have fabricated a large‐area sr‐In_2_S_3_ photoanode (15 cm^2^) and subsequently assessed its PEC IOR performance (**Figure** **5**a). As shown in Figure 5b, the In_2_S_3_ photoanode with a large area exhibited a substantially higher photocurrent of 22 mA at 1.23 V versus RHE, 56 times greater than the small area (0.11 cm^2^, 0.39 mA). Figure 5c demonstrated that large‐area sr‐In_2_S_3_ exhibits a relatively stable photocurrent density for IOR. Initial fluctuation in current density is attributed to the defects across the expanded surface area and the nonlinear diffusion of reactants and resultants on large‐scale photoanodes.^[^ ^70^, ^71^ ^]^ This implies that the high photocurrent and large area contributes to an increase in the production of economically viable products, thereby confirming the practical feasibility of our flame‐based surface reconstruction. All these findings prove that the flame‐based surface reconstruction enables the development of high‐performance sulfide‐based photoelectrode, thus enhancing the efficiency of solar‐to‐chemical fuel energy conversion.

## 3 Conclusion

In summary, we developed a flame‐based surface reconstruction method that enables the formation of conformal and uniform oxide layers and simultaneously generates S vacancies in the In_2_S_3_ photoanode. This flame‐based surface reconstruction significantly impacts the physicochemical properties of the In_2_S_3_ photoanode, reducing the charge recombination in the surface and bulk region and thus considerably enhancing photocurrent density and stability. Notably, the sr‐In_2_S_3_ photoanode achieved a significantly enhanced OER photocurrent density value of 8.5 and 9.5 mA cm^−2^ at 1.23 V versus RHE with and without a hole scavenger, which is one of the highest values compared to the previously reported sulfide‐based photoanodes to date. In addition, the sr‐In_2_S_3_ photoanode exhibited promising PEC IOR performance and stability. Our surface reconstruction strategy via a flame process successfully enhances charge collection properties, offering valuable insights for developing efficient sulfide‐based photoelectrodes in solar‐fuel generation devices.

## 4 Experimental Section

### Fabrication of In_2_S_3_ Photoanode

In_2_S_3_ photoanode was synthesized by a hydrothermal method. First, indium(III) chloride tetrahydrate (0.774 g, Sigma, 97%) and thiourea (0.306 g, Acros, 99%) were dissolved in 70 mL of deionized (DI) water under magnetic stirring for 2 h. Next, the precursor solution was poured into a Teflon‐lined stainless‐steel autoclave (100 mL capacity), and four pieces of the cleaned FTO substrates (2 cm × 3 cm) were immersed in the solution. The autoclave was sealed and heated to 160 °C for 12 h in an oven. After naturally cooling to 30 °C, the reddish In_2_S_3_ samples were thoroughly washed with DI water and air‐dried.

### Flame Treatment

Surface reconstruction was realized by flame treatment using flame surface processing equipment (iFLA‐500, Applied Plasma Inc.). The liquefied petroleum gas (LPG) and air flow rates were controlled to be 5.223 and 130 L min^−1^, respectively, yielding a fuel‐to‐oxygen equivalence ratio (*Φ*) 1.1. For the flame treatment, the In_2_S_3_ photoanode was quickly inserted into the flame cone and held for 10–40 s.

### Material Characterizations

The morphology and thickness of the In_2_S_3_ photoanodes were observed using SEM (IT500HR, Jeol). TEM (JEM‐3010, Jeol) was used to investigate the microstructure and compositions of In_2_S_3_. The crystalline structure of the In_2_S_3_ was investigated using XRD (D/MAX‐Ultima III, Rigaku). The absorbance spectra were obtained using an ultraviolet–visible–near infrared spectrophotometer (UV‐2600i, Shimadzu Scientific). The X‐ray photoelectron spectroscopy was employed to confirm the surface chemical states and chemical bonding changes of the In_2_S_3_. PL and time‐resolved photoluminescence decay spectra were investigated using a spectrophotofluorometer with time‐correlated single photon counting (Fluorolog 3, Horiba Scientific) and an excitation wavelength (*λ*_ex_ = 374 nm). Contact angle meter (Phoenix 300, SEO) was employed to confirm the surface wettability of In_2_S_3_.

### PEC Measurements

The PEC performance of In_2_S_3_ photoanode was measured using a potentiostat (Model SP‐200; Biologic) in a three‐electrode configuration (a Ag/AgCl (3 m KCl) reference electrode and a Pt‐wire counter electrode) under the illumination of simulated solar light (AM 1.5 G, 100 mW cm^−2^). Before the measurement, the solar simulator intensity was calibrated with a reference silicon solar cell. The illuminated area of the working In_2_S_3_ photoanode was 0.11 cm^2^, defined by a mask. 0.5 m Na_2_SO_4_ and 0.5 m Na_2_SO_3_ solutions were used as the electrolyte and hole scavenger, respectively. The *J*–*V* curve was recorded by linear sweep voltammetry measurements at a scan rate of 50 mV s^−1^. According to the Nernst equation, the potential versus the RHE was converted by the equation: *V*_RHE_ = *E*°_Ag/AgCl_ (0.210 V) + 0.059 × pH + *V*_Ag/AgCl_. The incident photon‐to‐current conversion efficiency (IPCE) was measured at 1.23 V versus RHE using IPCE equipment (HS Technologies, PE IPCE). APCE was calculated using the following equation: APCE(λ) = IPCE(λ)/*A*(λ), where *A* represents optical absorption. The PEIS measurements were conducted in the frequency range from 100 kHz to 1 Hz. The IOR was measured in 0.5 m Na_2_SO_4_ + 0.1 m KI (Sigma, ≥99%).

## Conflict of Interest

The authors declare no conflict of interest.

## Supporting information

Supporting Information

## Data Availability

The data that support the findings of this study are available from the corresponding author upon reasonable request.
