# Supplementary material for: Rapid Surface Reconstruction of In2S3 Photoanode via Flame Treatment for Enhanced Photoelectrochemical Performance
Source: Adv Mater. 2024 May 30;37(26):2403164. doi: 10.1002/adma.202403164 (PMC12232229; doi:10.1002/adma.202403164)
Supplement: Supplementary file 1 — Supporting Information [file ADMA-37-2403164-s001.docx]

**Supporting information**

**Rapid Surface Reconstruction of In_2_S_3_ Photoanode via Flame Treatment for Enhanced Photoelectrochemical Performance**

Yoo Jae Jeong,^1,2,a^ Runfa Tan,^1,2,a^ SeongSik Nam,^3,5,a^ Jong Ho Lee,^1,2^ Sung Kyu Kim,^4^ Tae Gyu Lee,^2^

Seong Sik Shin,^3,5,^* Xiaolin Zheng,^6,^* and In Sun Cho^1,2,^*

*^1^Department of Energy Systems Research, Ajou University, Suwon, 16499, Republic of Korea*

*^2^Department of Material Science & Engineering, Ajou University, Suwon, 16499, Republic of Korea*

*^3^Department of Nano Engineering | Department of Nano Science and Technology | SKKU Advanced Institute of Nanotechnology (SAINT), Sungkyunkwan University, Suwon, 16419, Republic of Korea*

*^4^Department of Nanotechnology and Advanced Materials Engineering, Sejong University, Seoul, 05006, Republic of Korea*

*^5^SKKU Institute of Energy Science and Technology (SIEST),* *Sungkyunkwan University, Suwon, 16419, Republic of Korea*

*^6^Department of Mechanical Engineering, Stanford University, Stanford, CA 94305, USA*

^a^These authors contributed equally

***Corresponding Authors:**

**Seong Sik Shin,** E-mail: [sss85@skku.edu](mailto:sss85@skku.edu)

**Xiaolin Zheng,** E-mail: [xlzheng@stanford.edu](mailto:xlzheng@stanford.edu)

**In Sun Cho,** Tel: +82-31-219-2468; Fax: +82-31-219-1613; E-mail: [insuncho@ajou.ac.kr](mailto:insuncho@ajou.ac.kr)

**Experimental method**

The charge transport (η_transport_) and transfer (η_transfer_) efficiencies were obtained using the following equations:

*η*_transport =_ $\frac{J_{{Na}_{2}SO_{3}}}{J_{abs}}$ , *η*_transfer =_ $\frac{J_{H_{2}O} or J_{IOR}}{J_{{Na}_{2}SO_{3}}}$

where $J_{H_{2}O}$ and $J_{{Na}_{2}SO_{3}}$ are the recorded photocurrent densities in the presence or absence of a hole scavenger (Na_2_SO_3_), respectively. $J_{IOR}$ is measured in the resence of potassium iodide (KI). The J_abs_ is the integrated photon flux value from absorbance spectra. Applied bias Photon-to-Current Efficiency (ABPE) can be calculated by the following equation:

$$ABPE \left( \% \right)= \frac{(J_{ph}\times\left( 1.23-V \right))}{P_{total}}$$

where J_ph_ is the photocurrent density marked out J-V plots, V is applied potential, and P_total_ represents input light power density (100 mW/cm^2^).

Mott-Schottky (MS) analysis was conducted under dark conditions with a frequency of 1 kHz, and subsequently depicted utilizing the following correlation:

$$\frac{1}{C^{2}}=\frac{2}{\varepsilon_{0}\varepsilon_{r}A^{2}eN_{D}}(V-V_{fb}-\frac{\mathrm{kT}}{e})$$

where C is the space charge capacitance, $N_{D}$ is the carrier density (donor), $\varepsilon_{0}$ is the permittivity of vacuum (8.854 × ${10}^{-19}$ F$m^{-1}$), $\varepsilon_{r}$ is the dielectric constant, $A$ is the surface area of the film (0.11 cm^2^), V is the applied potential, $V_{fb}$ is the flat band potential, $k$ is the Boltzmann constant (1.381 × ${10}^{-23}$ J$K^{-1}$), $e$ is the electron charge (1.602 x ${10}^{-19}$ C), and $T$is the absolute temperature (K).

***Gas chromatography and Faradaic efficiency measurements***

The gases produced from both the photoanode, and the Pt counter electrode were directly measured by applying a bias of 0.6 V_RHE_ in a three-electrode PEC cell. This cell was connected to a gas chromatograph (YL6500GC, Young In Chromass, South Korea) equipped with a pulsed discharge detector (PDD), a 5 Å zeolite molecular sieve column, and helium (He) as the carrier gas. Before measurement, the PEC cell and electrolyte underwent thorough purging with helium gas to eliminate nitrogen (N_2_) and oxygen (O_2_) gases from the PEC cell. A mass flow controller (VIC-D210, MKP) was utilized to continuously purge helium gas at a flow rate of 60 sccm, and gas samples were automatically collected every 10 minutes by gas chromatography. All measurements were conducted at room temperature (25°C). Faradaic efficiency (H_2_) was determined using the following equation:

$$Faradaic efficiency \left( FE \right)=\frac{moles of H_{2}}{J_{ph}\times A\times t/n\times e\times N_{A}}$$

where J_ph_ is the current density (A/cm^2^), A is the area (cm^2^), t is the time (s), e is the elementary charge (1.602 × 10^−19^ C), n = 2 for H_2_, and N_A_ is Avogadro’s number (6.02 × 10^23^ mol^-1^).

Faradaic efficiency (I_3_^-^) was determined using the following equation:

$$Faradaic efficiency \left( FE \right)=\frac{C\times n\times F\times V}{Q}$$

where n is number of electron transfer for IOR (2), F represents Faraday constant (96485 C mol^−1^), C is mole concentration of I_3_^‑^ (mol L^−1^), V is volume of electrolyte (L), and Q is total charge transfer in the IOR (C).

**Table S1.** Summary of photocurrent density (at 1.23 V vs. RHE) of sulfide-based photoanodes (In_2_S_3_, SnS_2_, CdIn_2_S_4_, and ZnIn_2_S_4_). *The J_ph_ values were roughly obtained from J-V curves in the corresponding paper.^[1–20]^


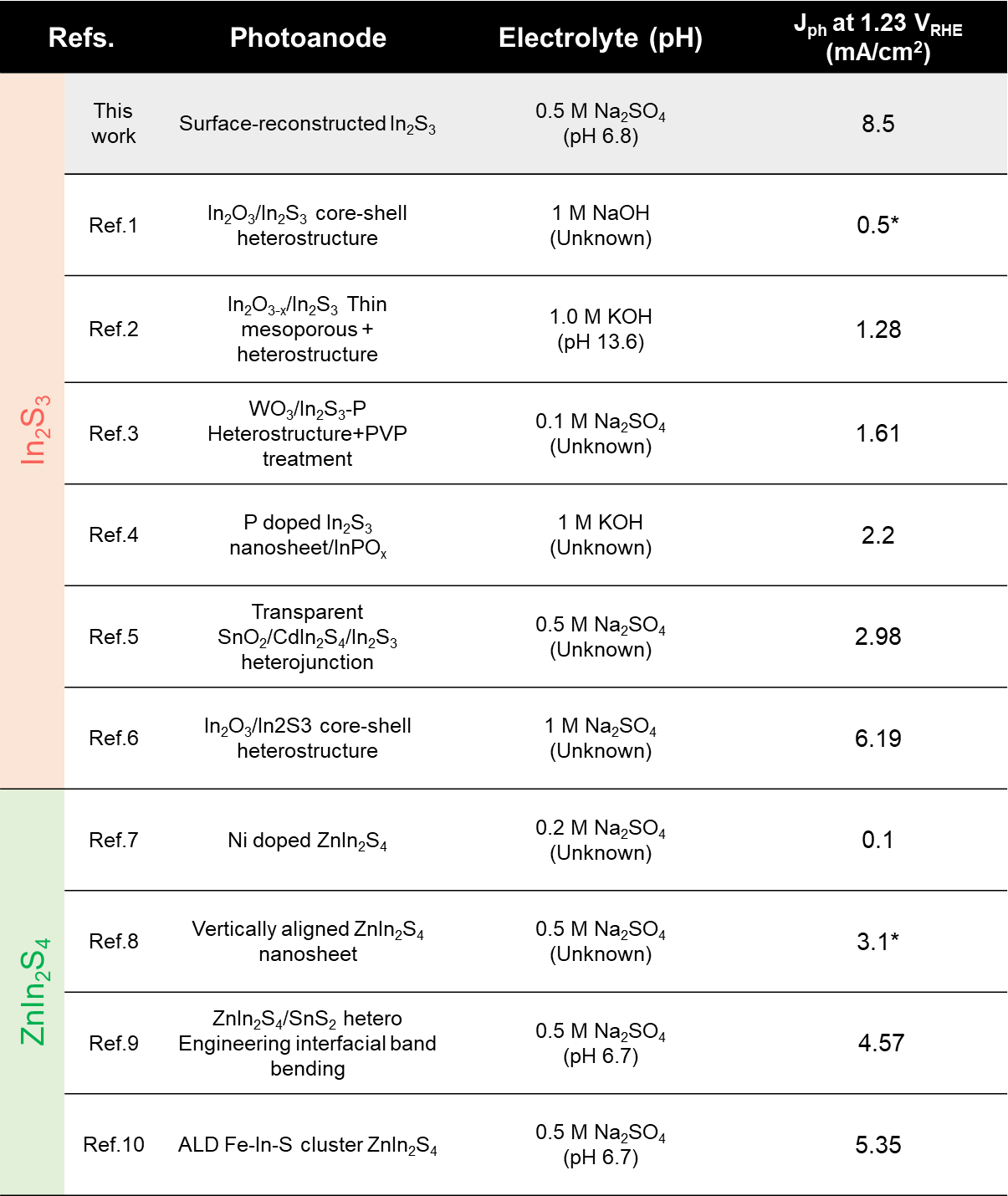


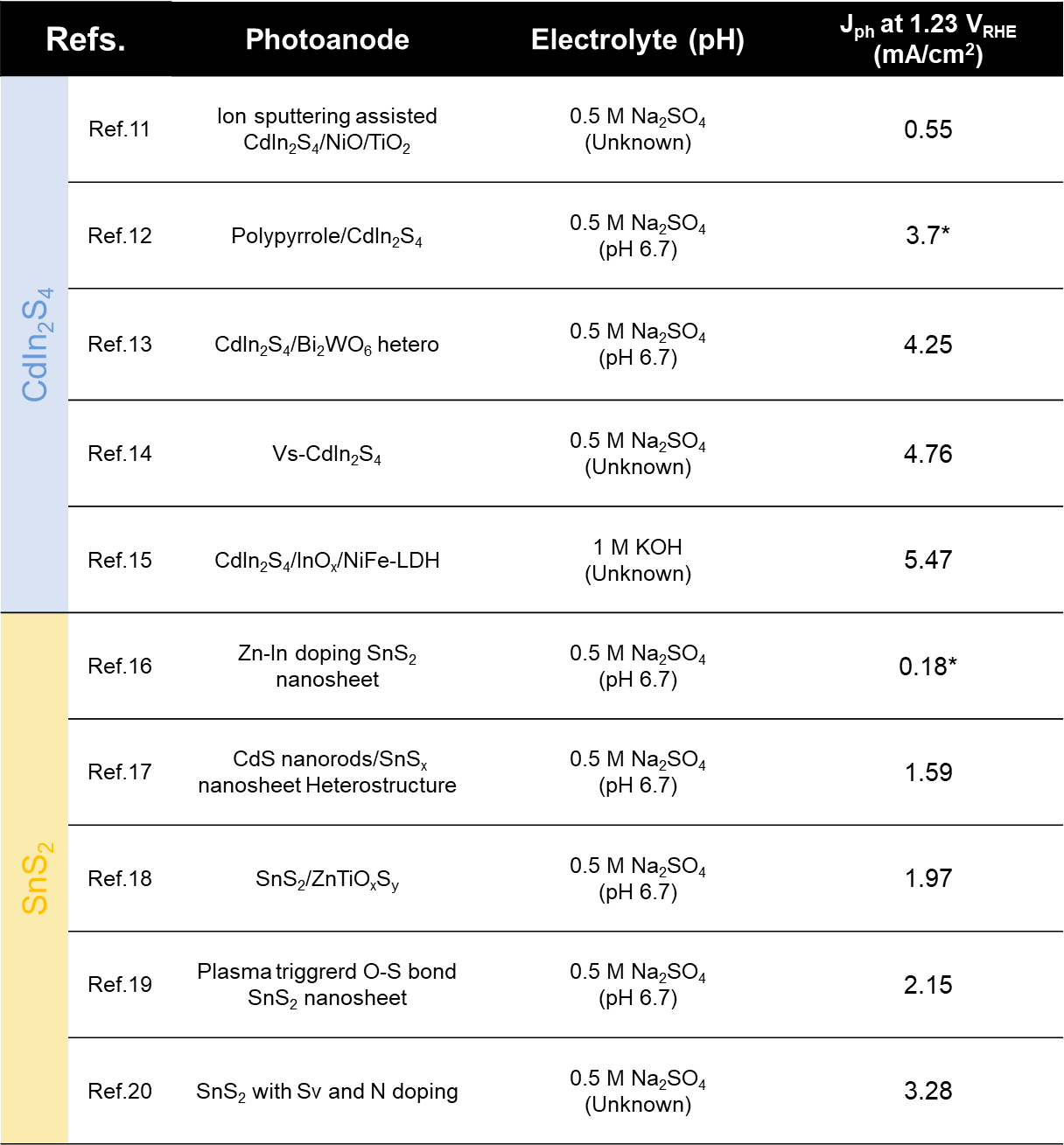


**Table S2.** Calculated carrier lifetime and Exponential decay-fitted parameters for In_2_S_3_ and sr-In_2_S_3_ photoanodes. The results were fitted most reasonably via a four-exponential decay model: I(t) = A_1_exp(-t/τ_1_) + A_2_exp(-t/τ_2_) + A_3_exp(-t/τ_3_) + A_4_exp(-t/τ_4_), where τ_1_, τ_2_, τ_3_, and τ_4_ are the decay times, and A_1_, A_2_, A_3_ and A_4_ are the corresponding amplitudes. The average carrier lifetimes τave were also calculated by using the equation: τ_ave_ = A_1_τ_1_ + A_2_τ_2_ + A_3_τ_3_ + A_4_τ_4_.


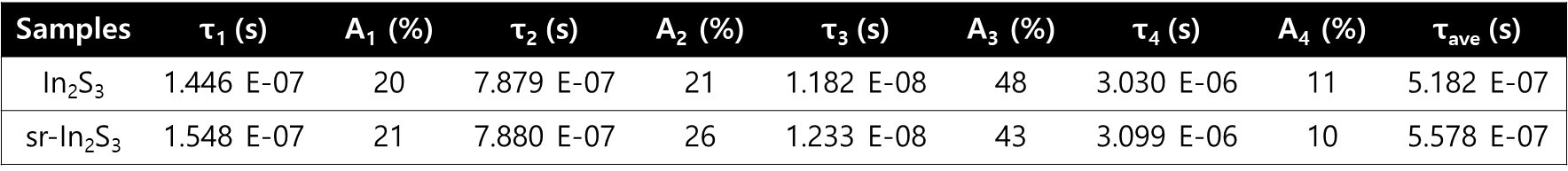


**
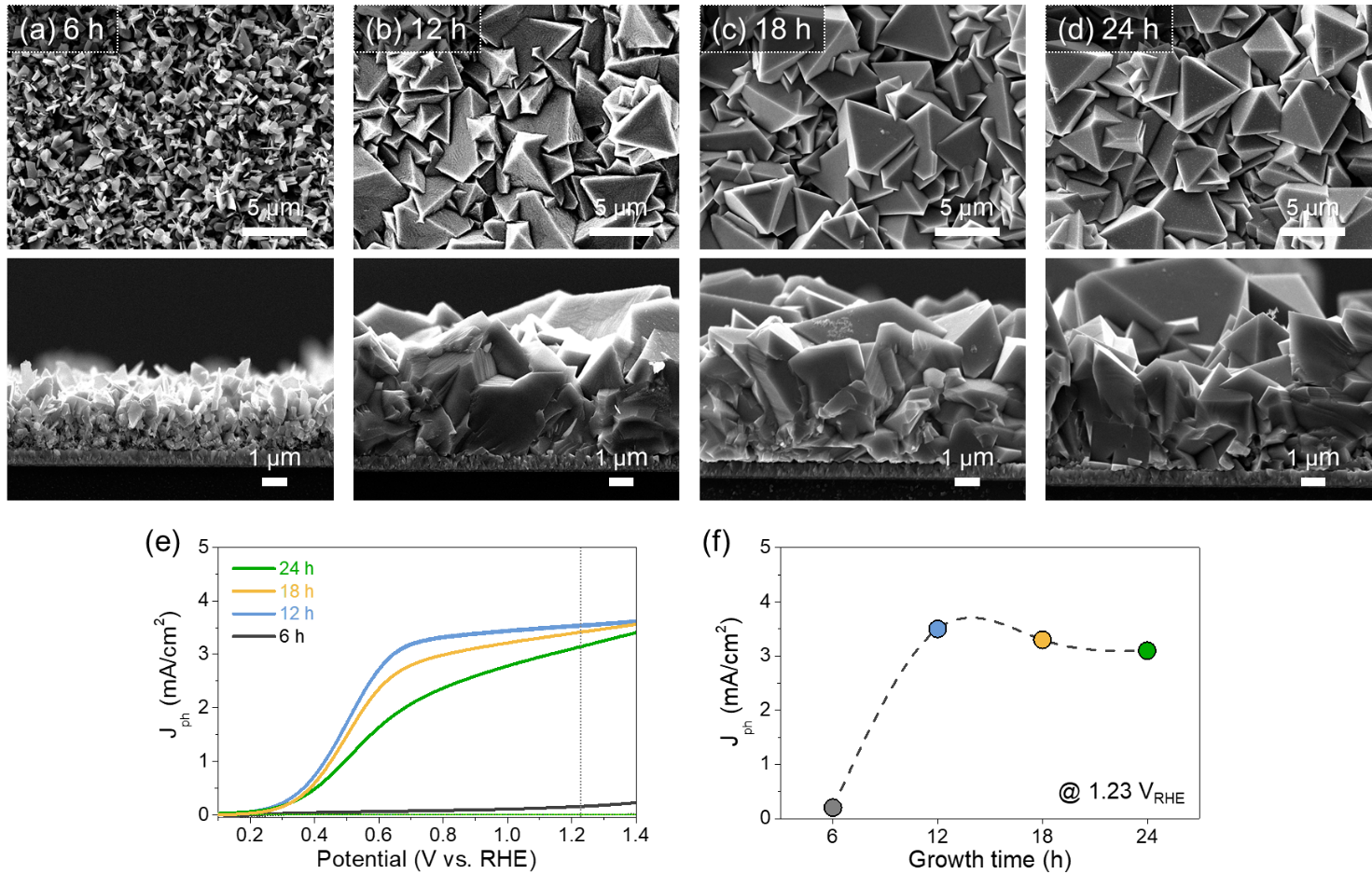
**

**Figure S1. Effect of hydrothermal growth time on the morphology and photocurrent density values (J_ph_).** SEM images of In_2_S_3_ photoanode grown at (a) 6 h, (b) 12 h, (c) 18 h, and (d) 24 h. (e) J-V curves. (f) Photocurrent density value (measured at 1.23 vs. RHE) vs. Growth time.

**
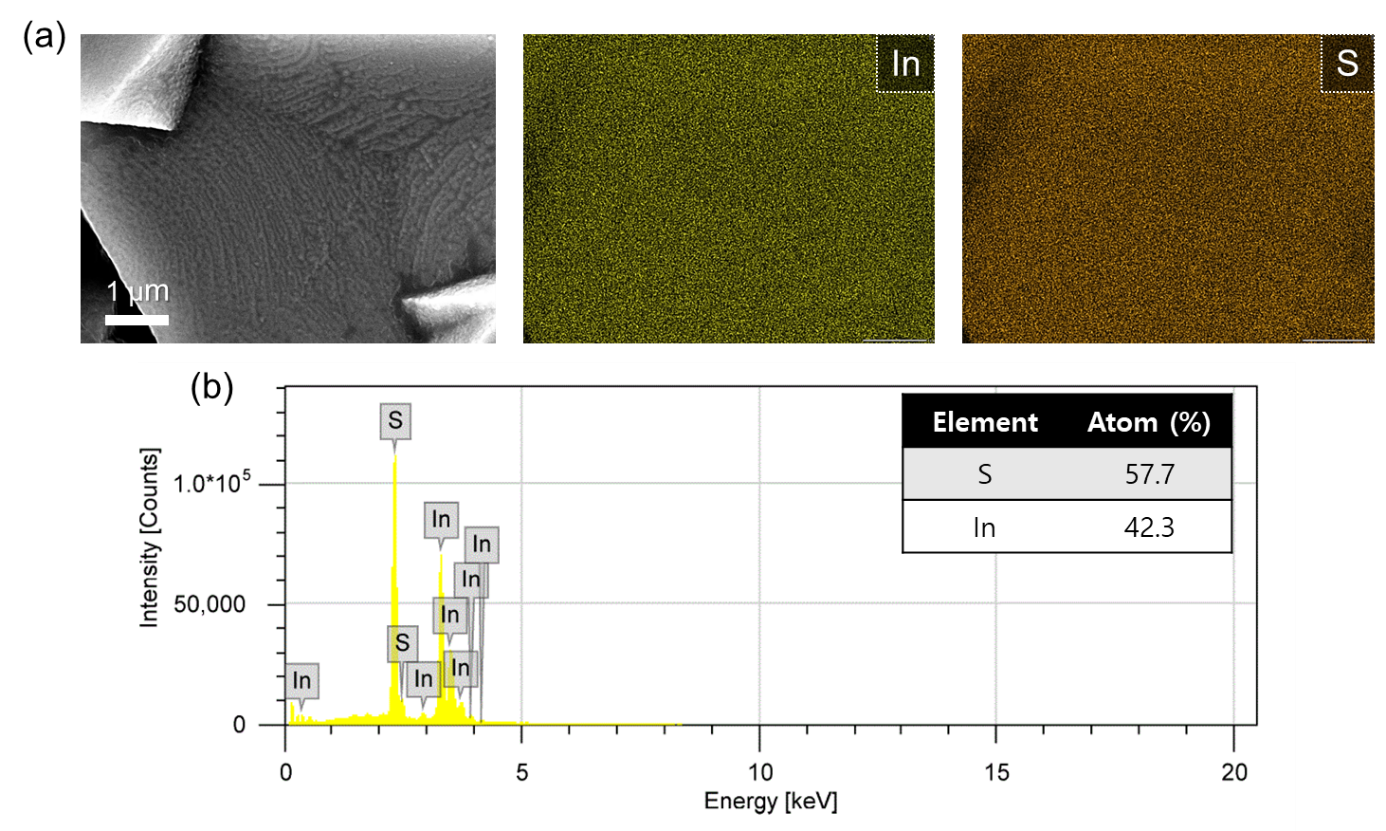
**

**Figure S2.** **EDS mapping results.** (a) Top-view EDS elemental mapping images and (b) EDS spectrum of In_2_S_3_ photoanode. The In_2_S_3_ photoanode was synthesized at 160 ℃ for 12 h. The In: S atomic ratio of In_2_S_3_ photoanode is about 2:3, indicating the successful synthesis of In_2_S_3_ photoanode.


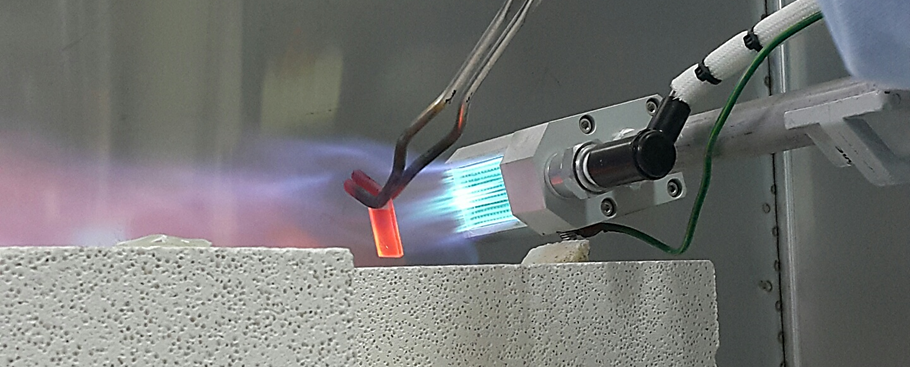


**Figure S3.** Photograph of the flame treatment process.


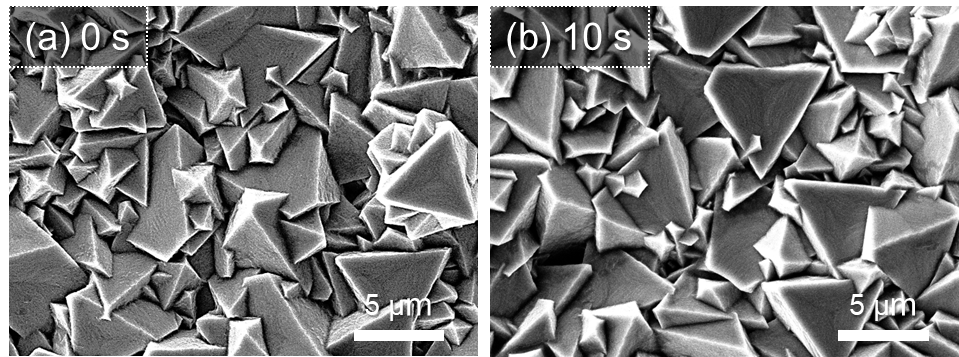


**Figure S4. Effect of flame treatment on the morphology of In_2_S_3_ photoanodes.** SEM top images of (a) pristine In_2_S_3_ and (b) flame-treated In_2_S_3_ (10 s).

**
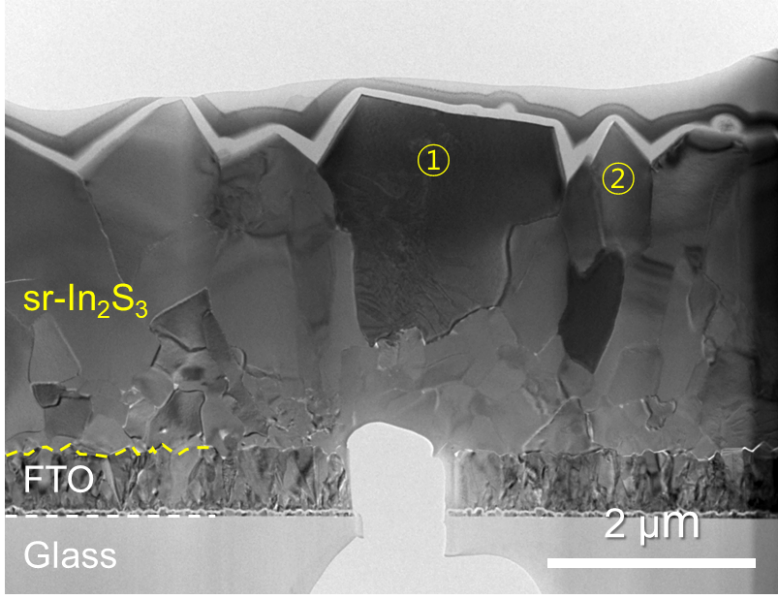
**

**Figure S5. Low-magnification TEM image of sr-In_2_S_3_ photoanode (10 s).** Two representative grains are observed (i.e., ① square and ② pyramid shaped-grains).


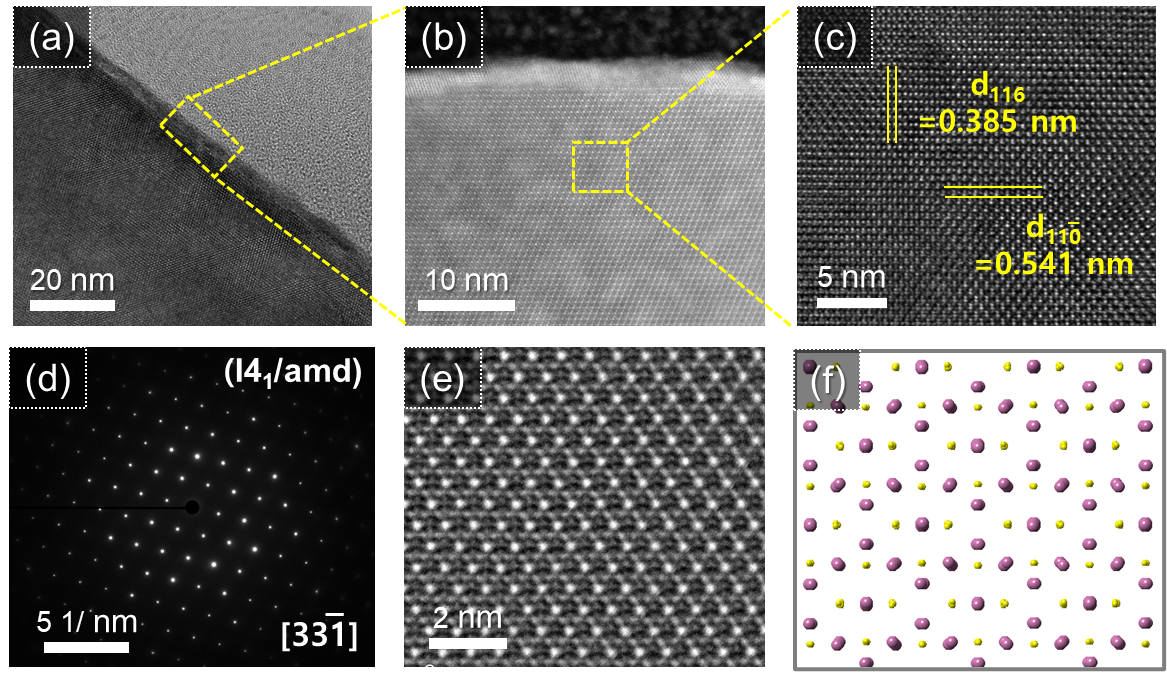


**Figure S6. TEM analysis of sr-In_2_S_3_ photoanode at square-shaped grain.** (a) TEM image. (b) High-angle annular dark-field scanning transmission electron microscopy (HAADF-STEM) image. (c) High-resolution (HR) TEM image. (d) Selected area electron diffraction (SAED) pattern. (e) STEM image. (f) Atomic structure of In_2_S_3_: zone axis=[33-1]. Purple and yellow spheres are In and S, respectively.


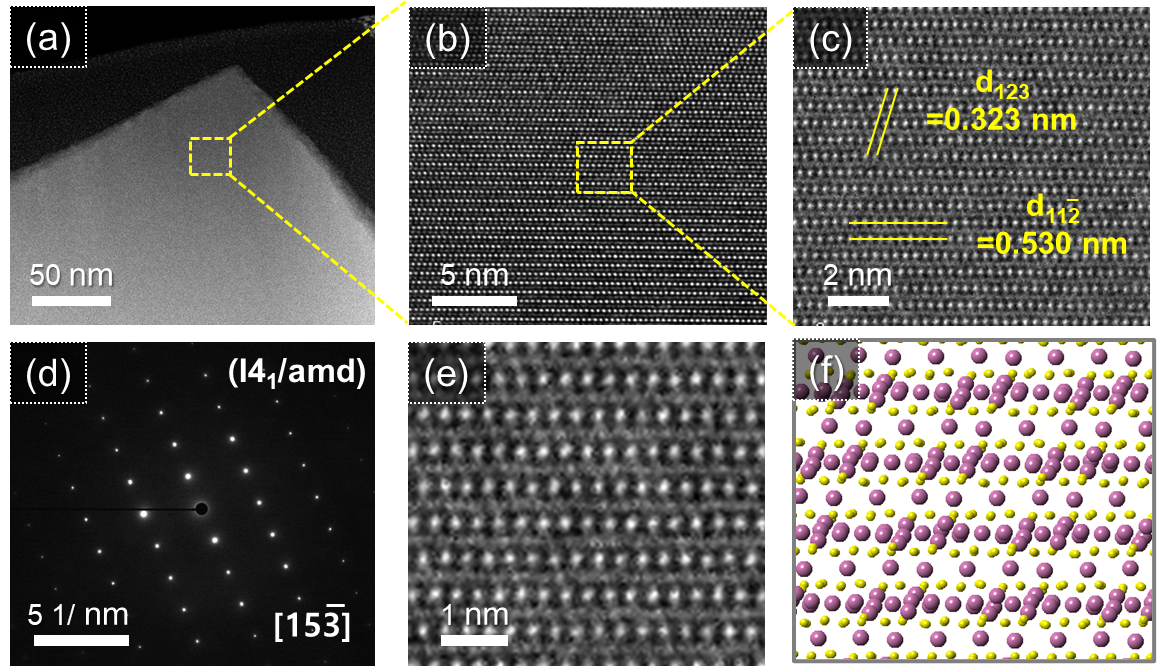


**Figure S7.** **TEM analysis of sr-In_2_S_3_ photoanode at pyramid-shaped grain.** (a) HADDF-STEM image. (b-c) TEM and HRTEM images. (d) SAED pattern. (e) STEM image. (f) Atomic structure of In_2_S_3_: zone axis=[15-3]. Purple and yellow spheres are In and S, respectively.


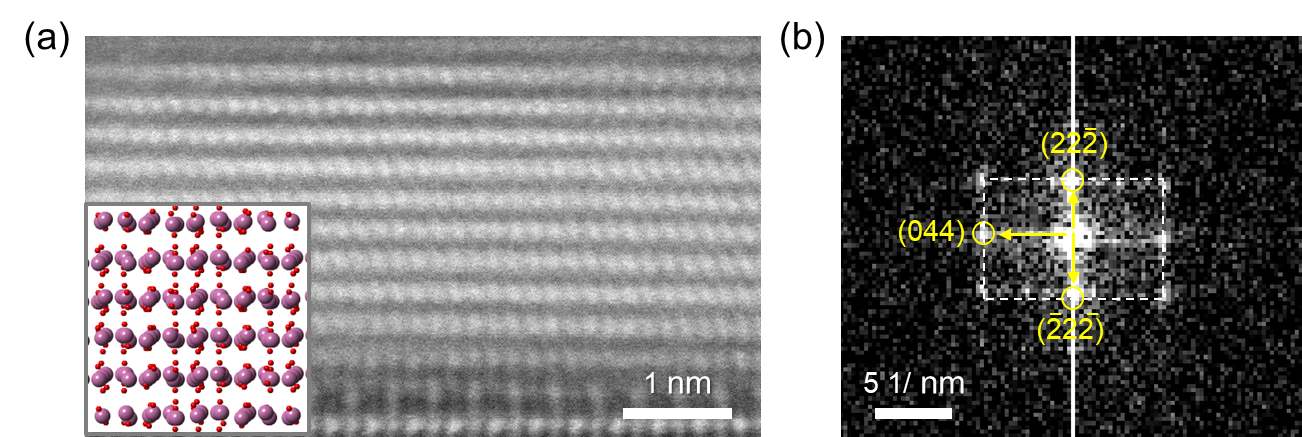


**Figure S8.** (a) High-magnification TEM image and (b) Fast Fourier transform (FFT) pattern of the crystalline In_2_O_3_ layer.


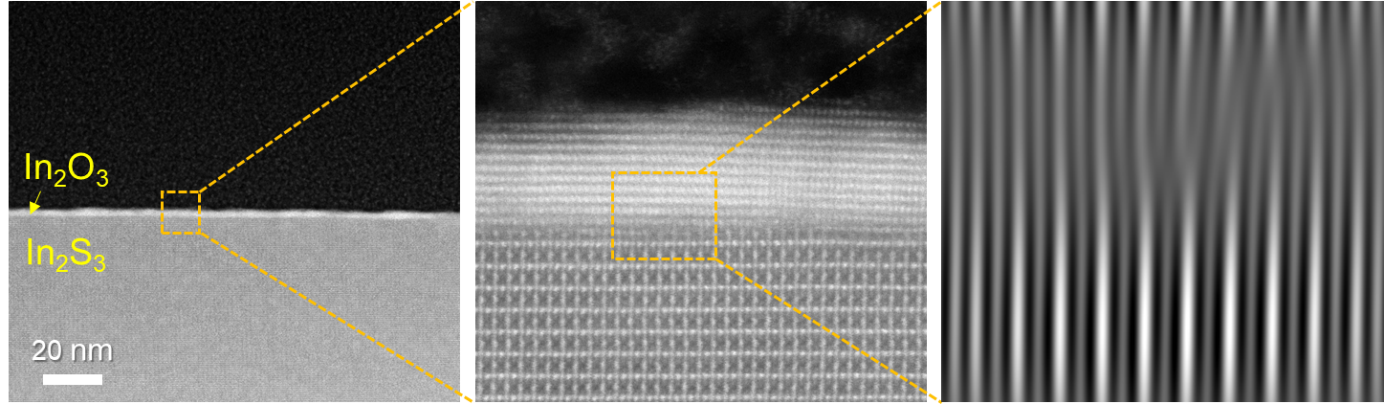


**Figure S9.** TEM and Fourier-filtered lattice images from the rectangular area of In_2_S_3_ and In_2_O_3_ interface in sr-In_2_S_3_ photoanode (10 s).


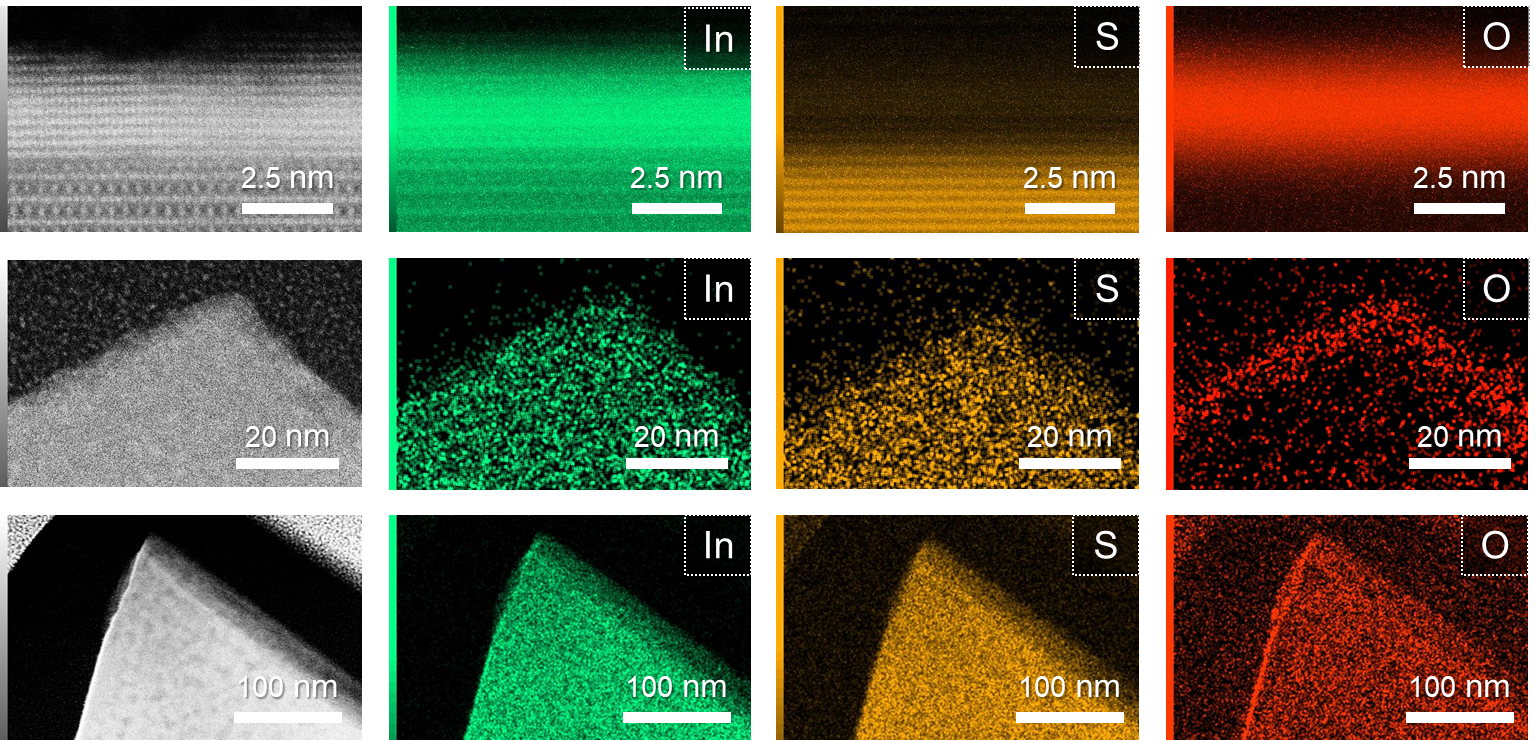


**Figure S10.** Energy dispersive spectroscopy (EDS) mapping of square-, and pyramid-shaped grains in sr-In_2_S_3_ photoanode (10 s).

**
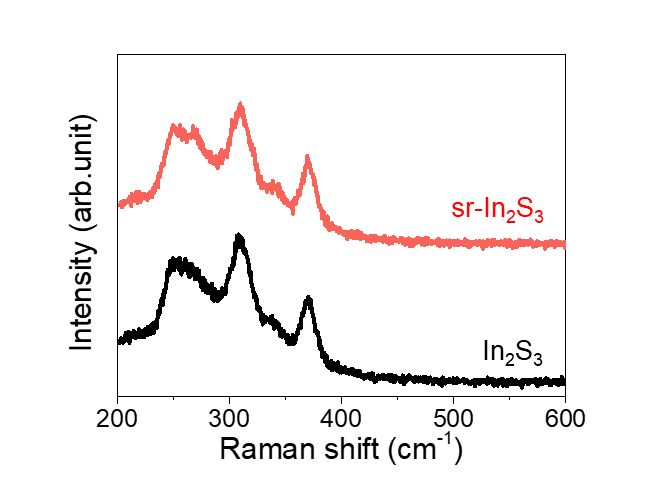
**

**Figure S11.** Raman analysis of sr-In_2_S_3_ and In_2_S_3_ photoanodes.

**
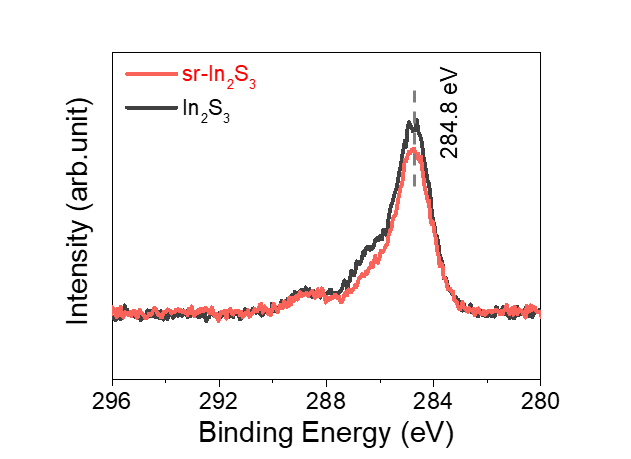
**

**Figure S12.** XPS spectra of C 1s obtained from sr-In_2_S_3_ and In_2_S_3_ photoanodes. The result indicates no incorporation of carbon species in the In_2_S_3_ photoanode due to the flame.


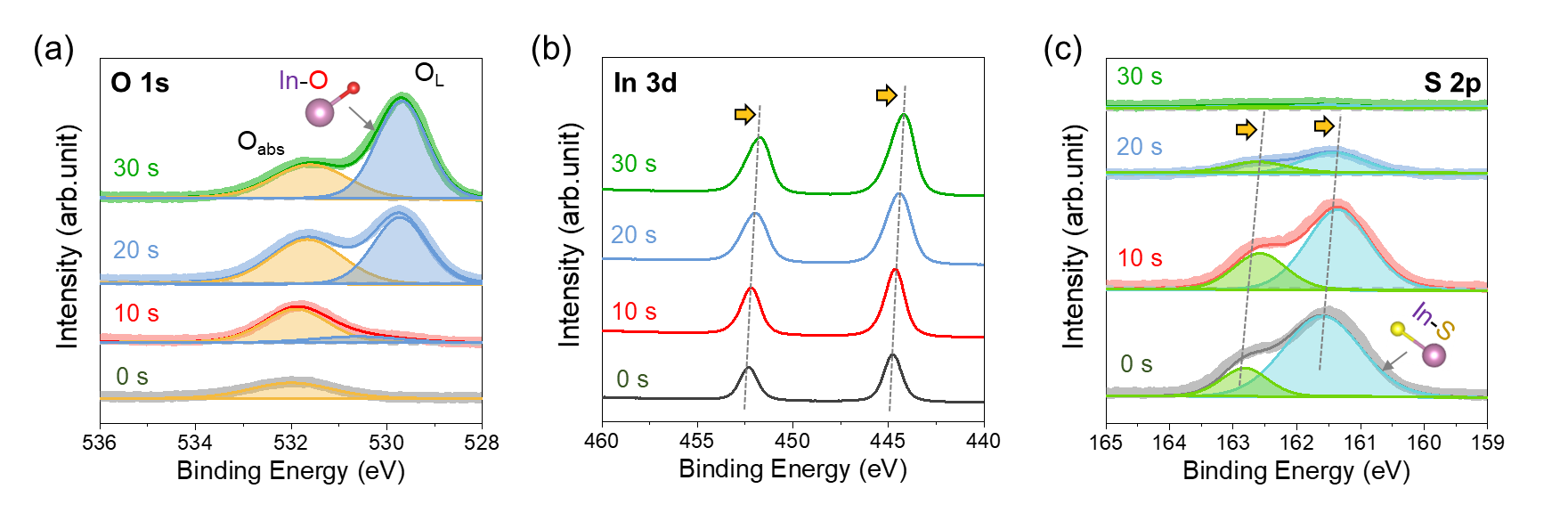


**Figure S13.** **Effect of flame duration times on the surface chemical states.** XPS spectra of (a) O 1s, (b) In 3d, and (c) S 2p.

**
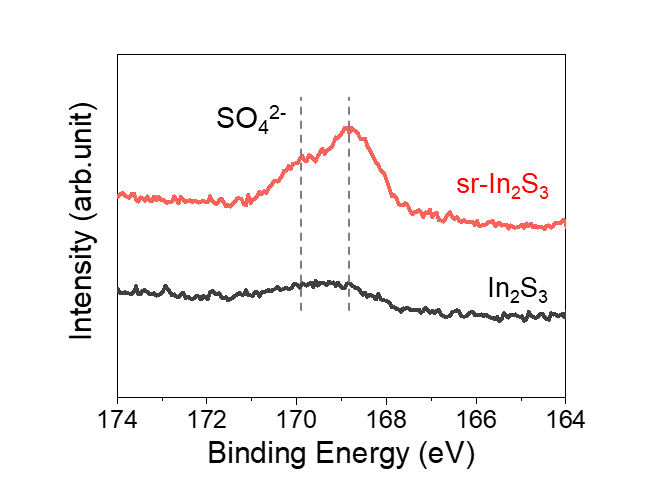
**

**Figure S14.** XPS spectra of S 2p from sr-In_2_S_3_ and In_2_S_3_ photoanodes.

**
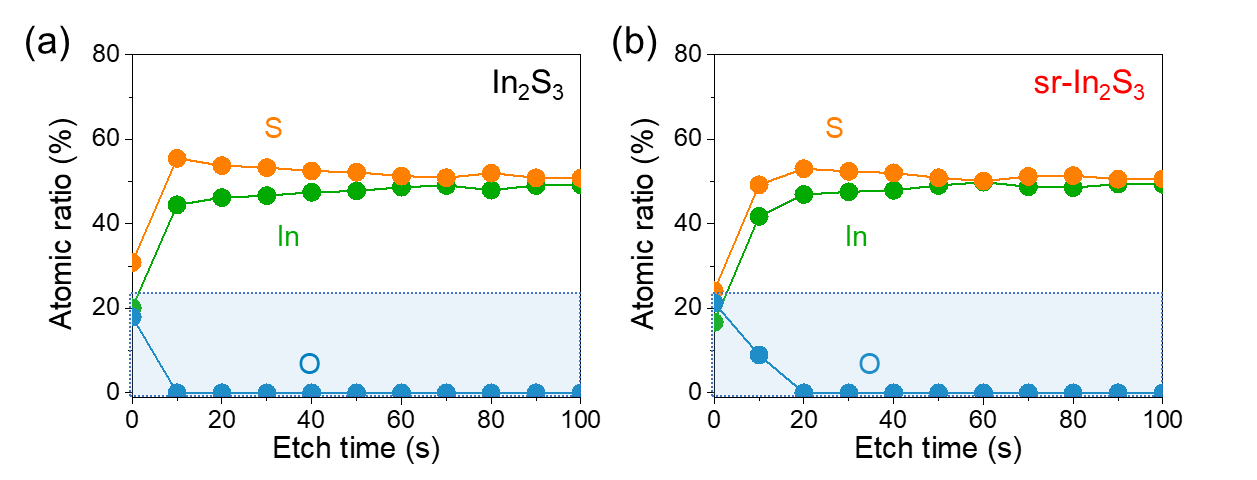
**

**Figure S15. XPS depth profile analysis.** Atomic ratios of (a) In_2_S_3_ and (b) sr-In_2_S_3_ photoanodes.

**
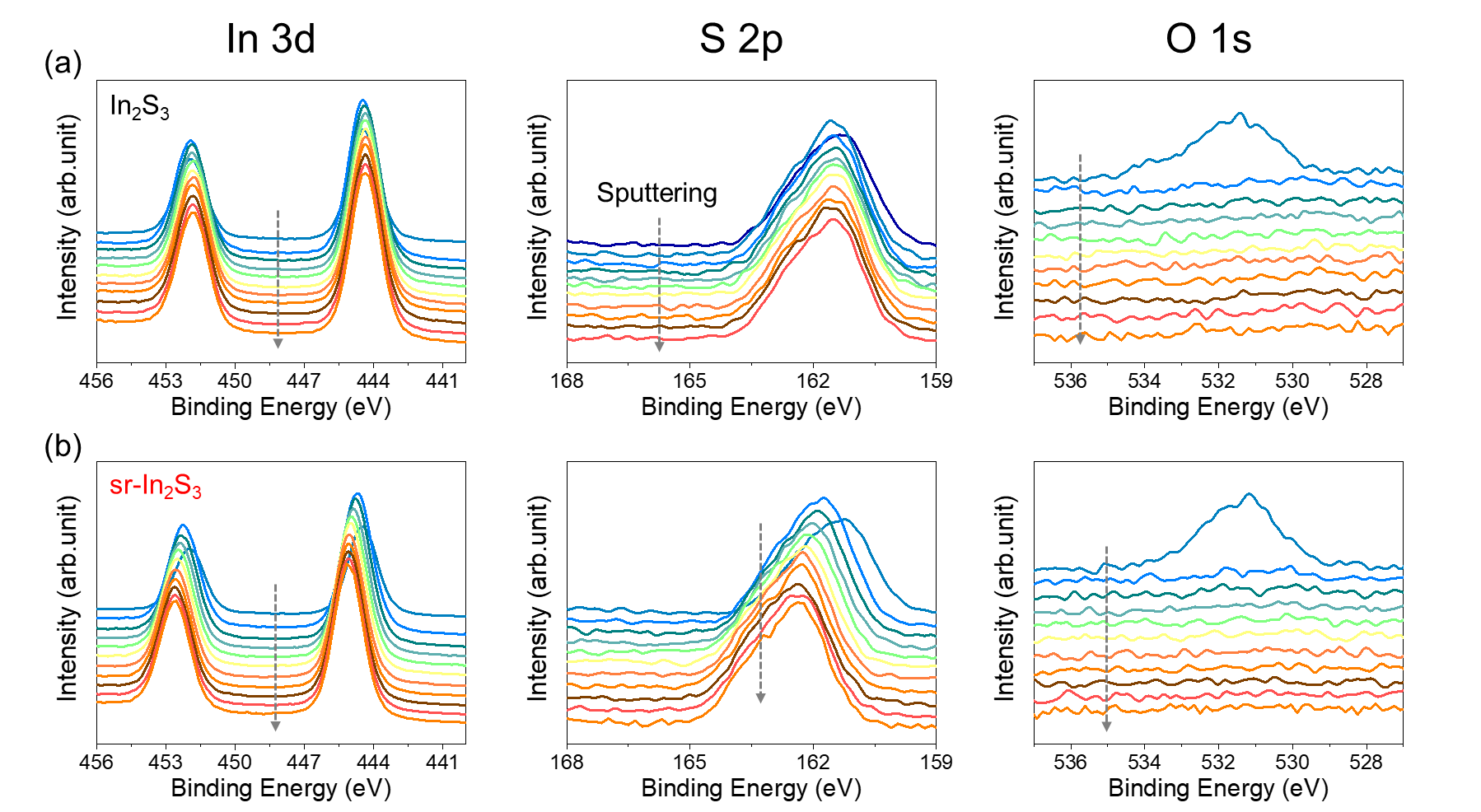
**

**Figure S16. XPS spectra obtained from depth profile analysis.** (a) In_2_S_3_ and (b) sr-In_2_S_3_ photoanodes.


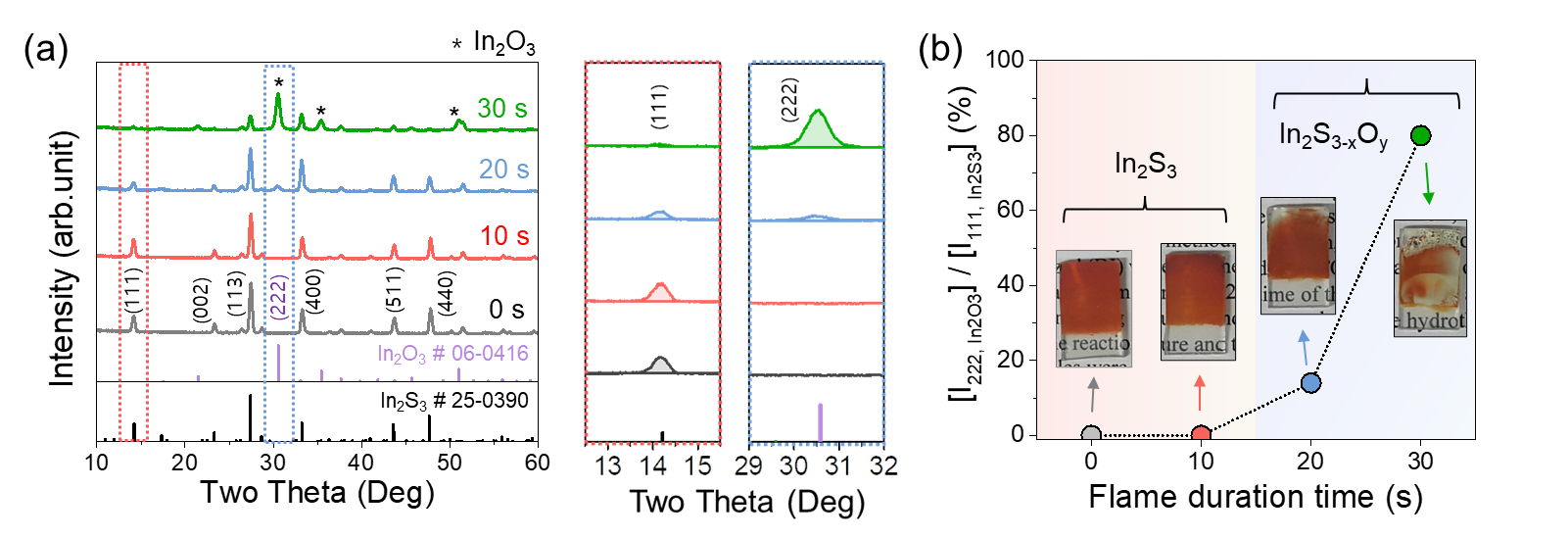


**Figure S17.** (a) XRD patterns. (b) Plane intensity ratios vs. flame treatment time (s) and show the photograph of the corresponding sr-In_2_S_3_ photoanode, respectively.

The relative XRD peak intensity of (111) In_2_S_3_ and (222) In_2_O_3_ was calculated, and the results are shown in **Fig. S17b**, together with the corresponding sample photographs. Both 0 and 10 s show identical sample color (dark brown) and a relative XRD peak intensity of 0%. However, for 20 s, the sample color changed to white, especially at the edge region. Also, it showed a 20% increase in the relative intensity. The 30 s displayed a white color (i.e., In_2_O_3_) in a large area with an 80% increase in the relative intensity.

**
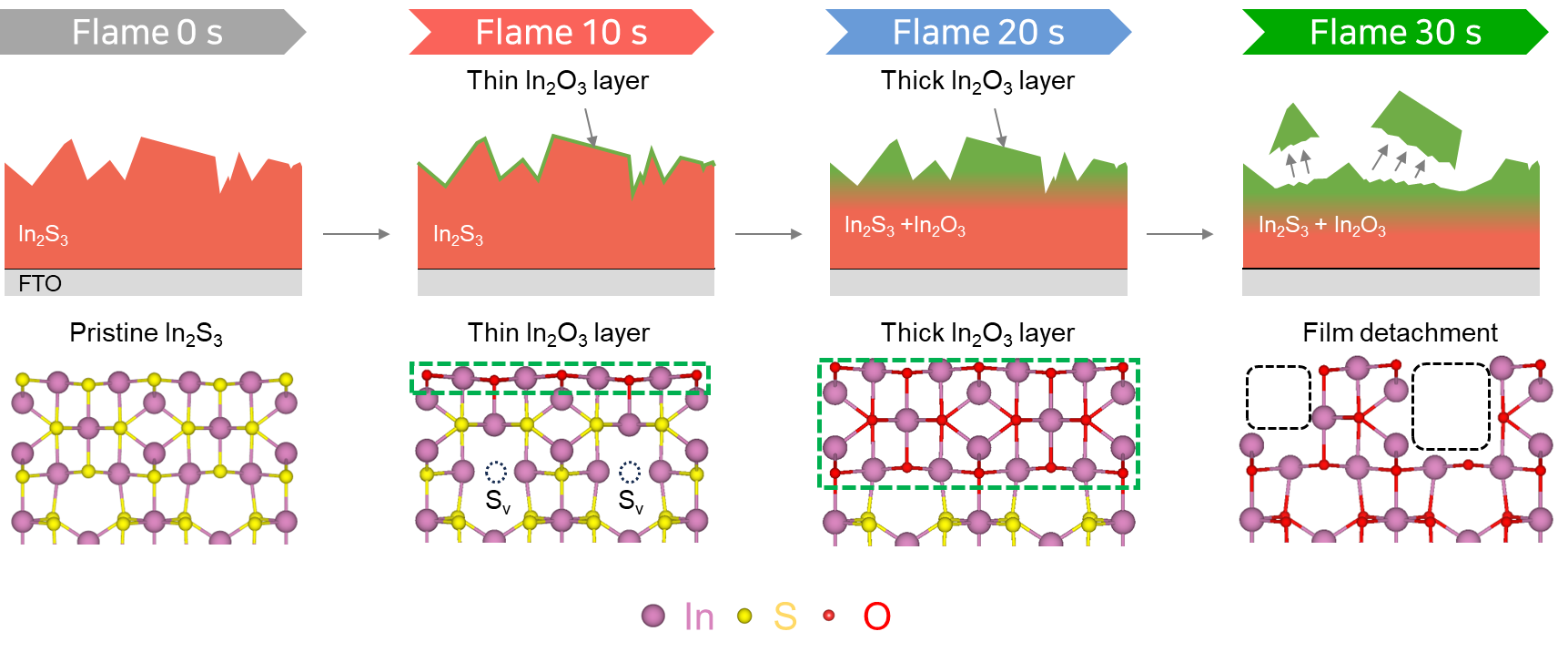
**

**Figure S18.** Overall surface reconstruction process of In_2_S_3_ photoanode with the flame duration time.

The overall surface reconstruction process of the In_2_S_3_ photoanode with the flame duration time was summarized. For the 10 s, the sr-In_2_S_3_ photoanode undergoes rapid surface reconstruction, leading to the formation of an In_2_O_3_ layer on the surface of the In_2_S_3_. This diffusionless surface oxidation process preserves the crystalline bulk In_2_S_3_ phase, effectively achieving surface passivation and introducing bulk S vacancies. Flame treatment lasting more than 20 s not only transforms the crystalline structures of In_2_S_3_ into In_2_O_3_ but also causes surface damage due to excess oxidation and thermal stress. Therefore, the surface reconstruction of the In_2_S_3_ photoanode can be achieved by simply controlling the flame treatment time, which simultaneously affects the formation of defects and crystalline structures in the In_2_S_3_ photoanode.


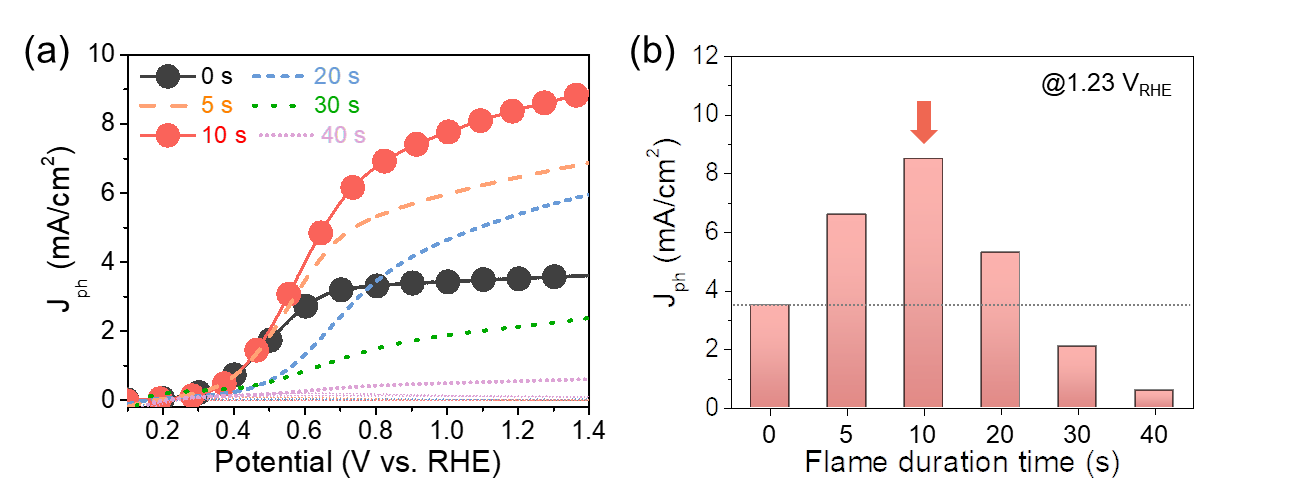


**Figure S19. Effect of flame duration time on In_2_S_3_ photoanodes.** (a) J-V curves. (b) J_ph_ (at 1.23 vs RHE) vs. Flame duration time.


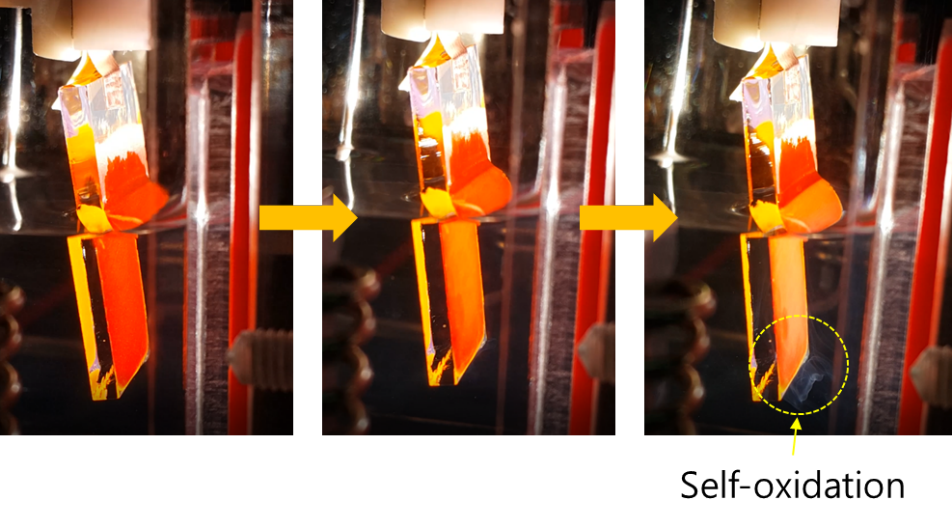


**Figure S20.** Photograph of the self-oxidation process (S^2-^ + 2h^+^ = S) on the In_2_S_3_ photoanode measured in the Na_2_SO_4_ electrolyte under 1-sun illumination.


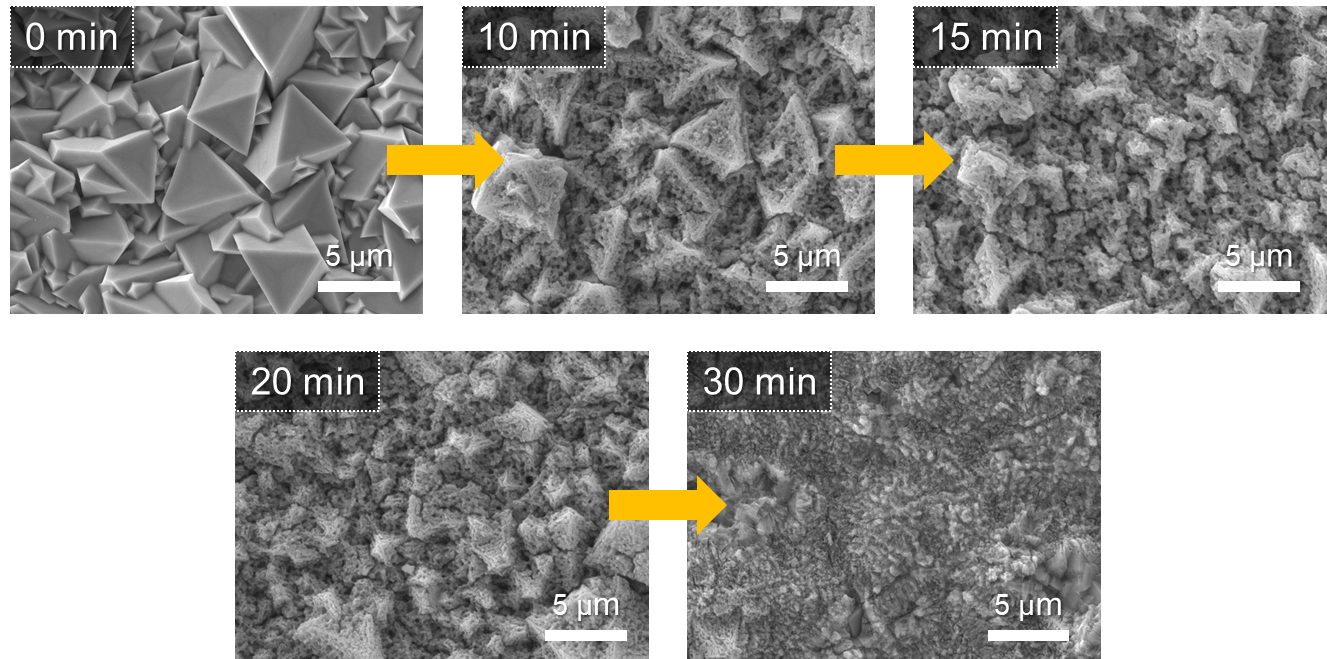


**Figure S21.** Photocorrosion behavior of the pristine In_2_S_3_ photoanode over PEC operation time.


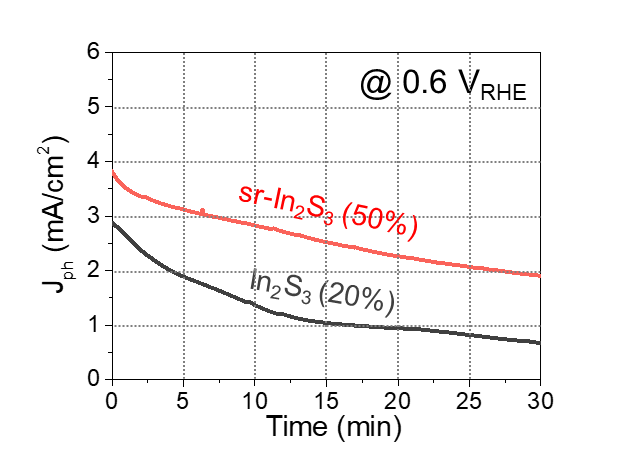


**Figure S22.** Photostability test of the sr-In_2_S_3_ and In_2_S_3_ photoanodes at 0.6 V_RHE_ in 0.5 M Na_2_SO_4_.


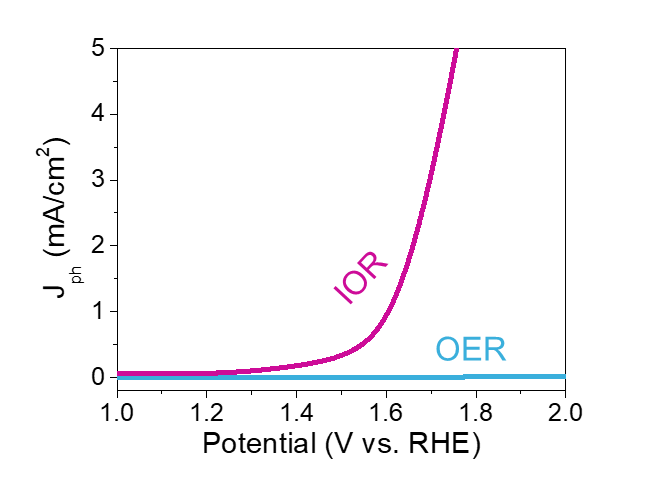


**Figure S23.** J-V curves of sr-In_2_S_3_ photoanode in the dark for IOR and OER.


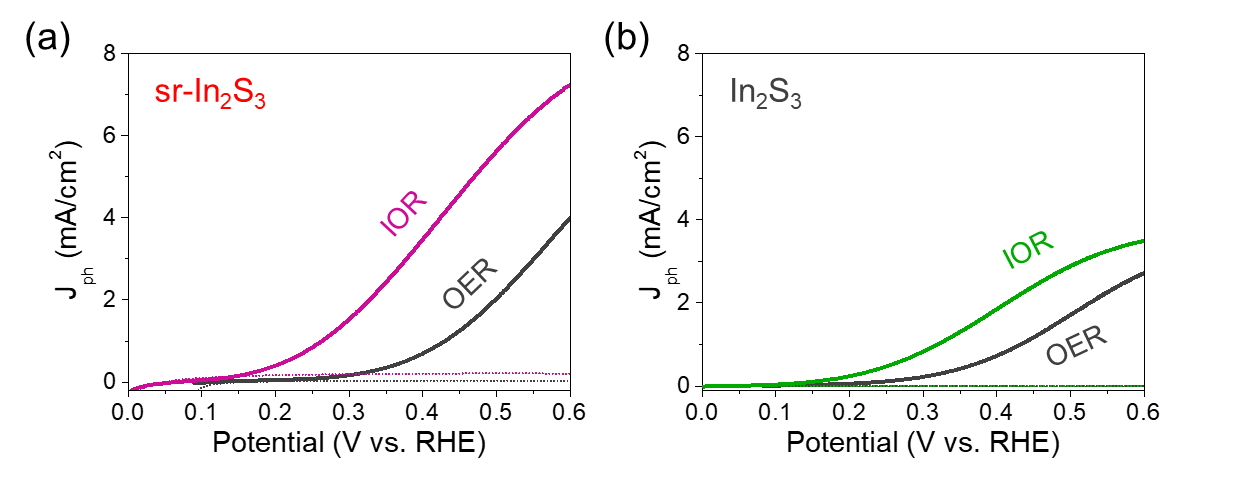


**Figure S24.** J-V curves of (a) sr-In_2_S_3_ and (b) In_2_S_3_ photoanodes measured in 0.5 M Na_2_SO_4_ with/without adding 0.1 M KI.


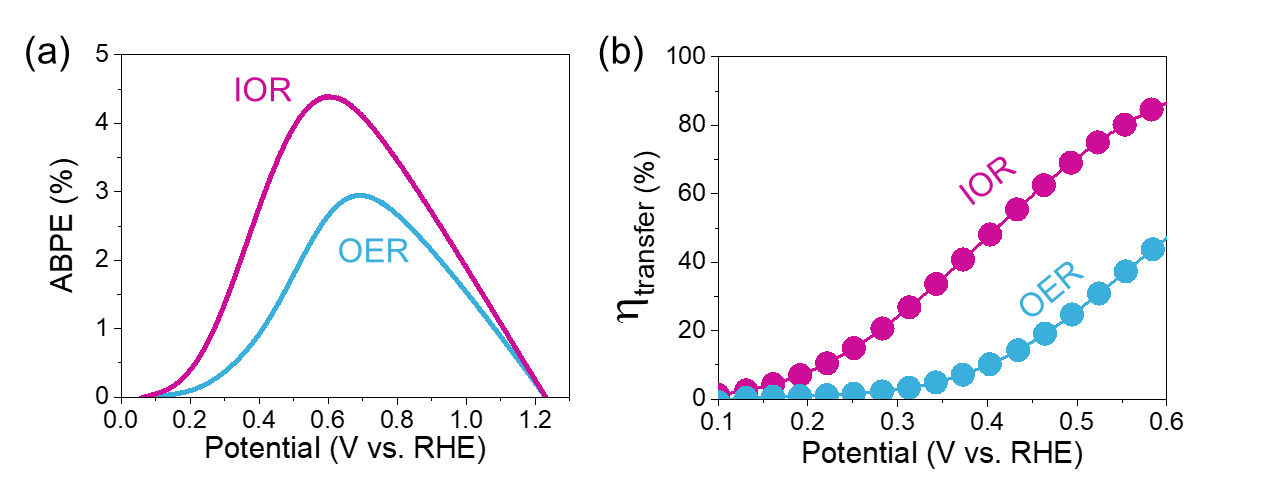


**Figure S25.** (a) ABPE curve and (b) Charge transfer efficiency (η_transfer_) of sr-In_2_S_3_ photoanode for IOR and OER.


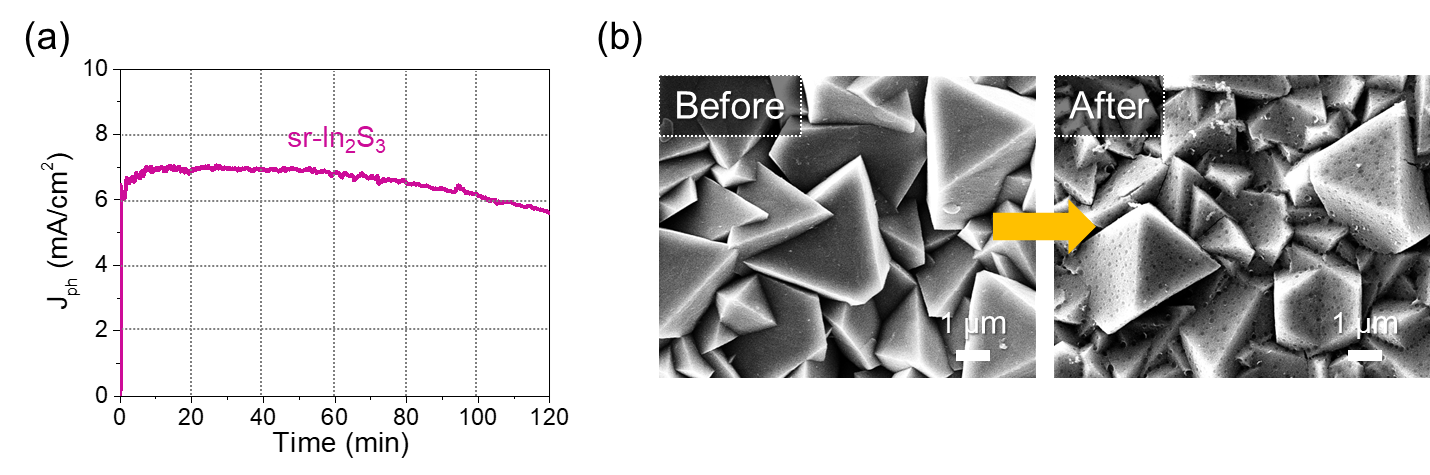


**Figure S26.** (a) Prolonged J-t measurement and (b) SEM images of sr-In_2_S_3_ photoanode before/after IOR measurement (80 min). After IOR measurement (80 min), SEM analysis shows the corrosion of sr-In_2_S_3_ photoanode itself, especially numerous pores and cracks. Despite faster IOR kinetics compared to OEC, the generated carriers may not be fully consumed in surface reactions. This results in the photocorrosion of sr-In_2_S_3_ photoanode, thus leading to performance degradation.

The initial photocurrent increase is attributed to the efficient adsorption of iodide molecules on the surface defect sites, i.e., oxygen vacancy from In_2_O_3_, allowing the enhanced charge transfer at the surface, eventually increasing the photocurrent.

**
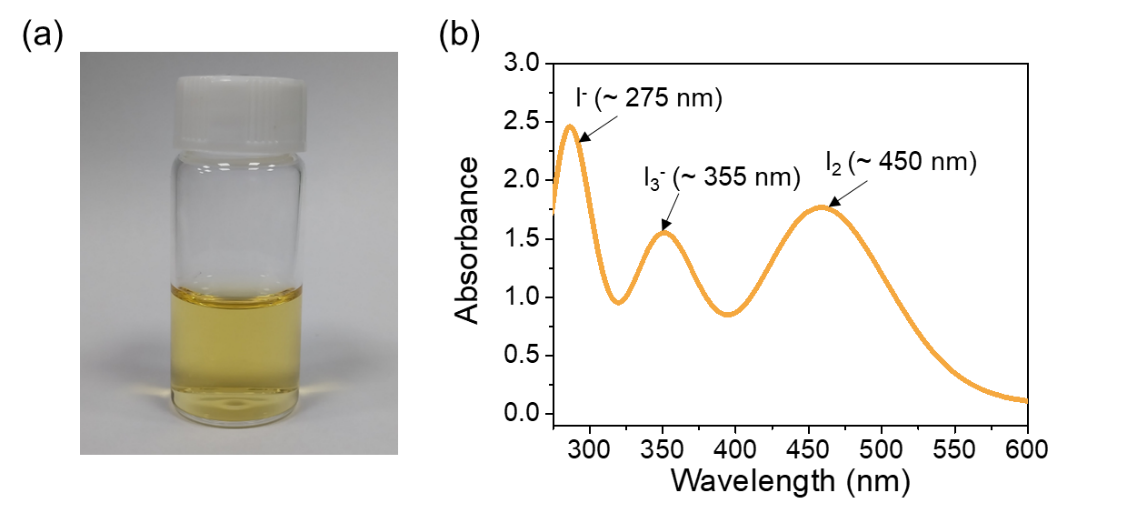
**

**Figure S27.** (a) Photograph and (b) UV-vis spectra of I_2_ reference solution.


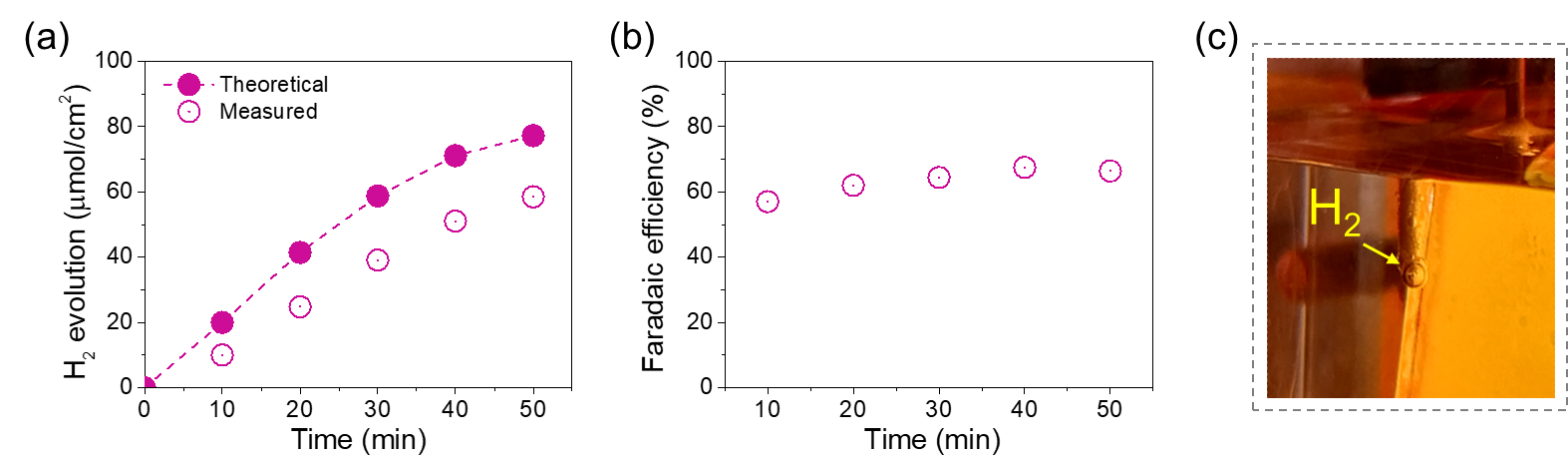


**Figure S28.** (a) H_2_ evolution, (b) Faradaic efficiency, and (c) a photograph of H_2_ evolution via iodide oxidation of sr-In_2_S_3_ photoanode. (measured at 0.6 V_RHE_)

According to the literature, platinum (Pt) can easily reduce triiodide, indicating that the back reactions of triiodide to iodide at the cathode are highly possible as long as a single-cell reactor is used. Therefore, an H-type cell consisting of a membrane to isolate the anodic and cathodic reactions should be employed to show a much higher FE of our sr-In_2_S_3_ photoanode. In addition, a flow cell and membrane system can enhance the FE by directing gas bubbles and isolating each electrode.^[21]^


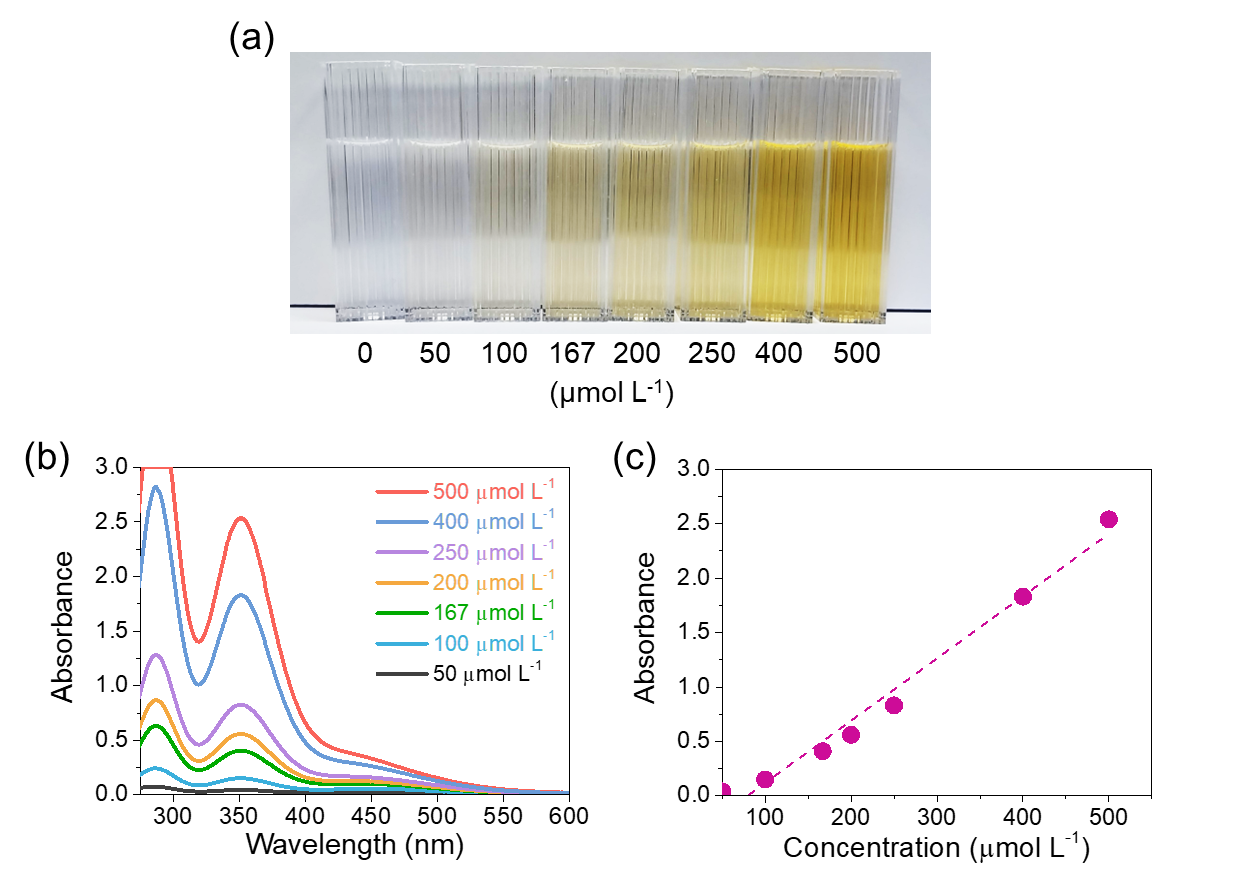


**Figure S29.** **Quantification of I_3_^-^ from calibration curve.** (a) Photograph of standard I_3_^-^ solution at different concentrations (Low concentration on the left to high concentration on the right. (b) UV-vis absorbance spectra obtained from standard I_3_^-^ solution at specific concentrations. (c) Calibration curve obtained from I_3_^-^ absorbance spectra at 355 nm. For the electrolyte (at a 1/10 dilution in **Figure 3g**), the absorption intensity at 353 nm is approximately 1.2, suggesting a concentration of 290.7 μmol L^-1^.


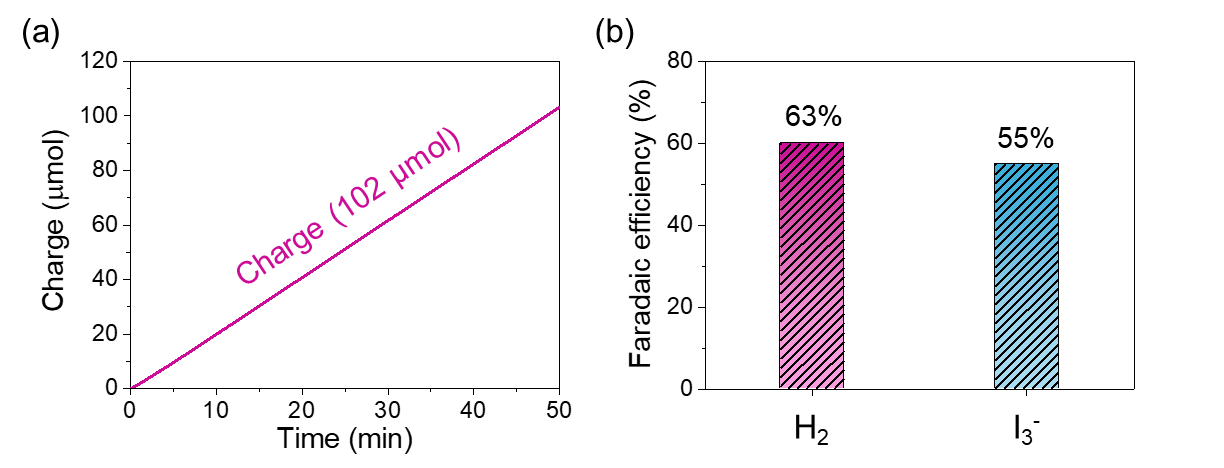


**Figure S30.** (a) Curve of the total electron amount obtained from integration of J-t curve. (b) Faradaic efficiency of H_2_ and I_3_^-^ evolutions.


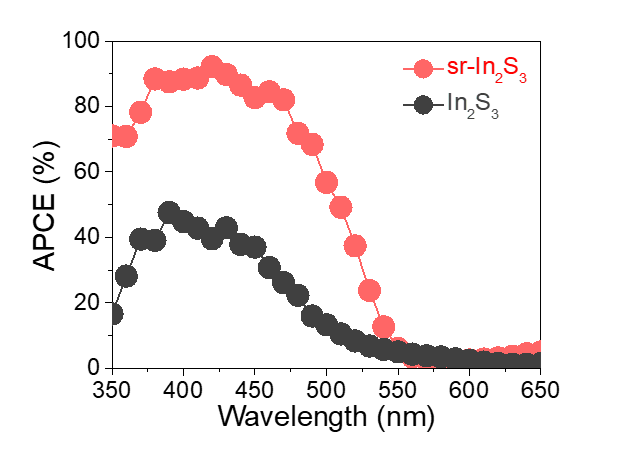


**Figure S31.** Absorbed photon-to-current efficiency (APCE) spectra of sr-In_2_S_3_ and In_2_S_3_ photoanodes.


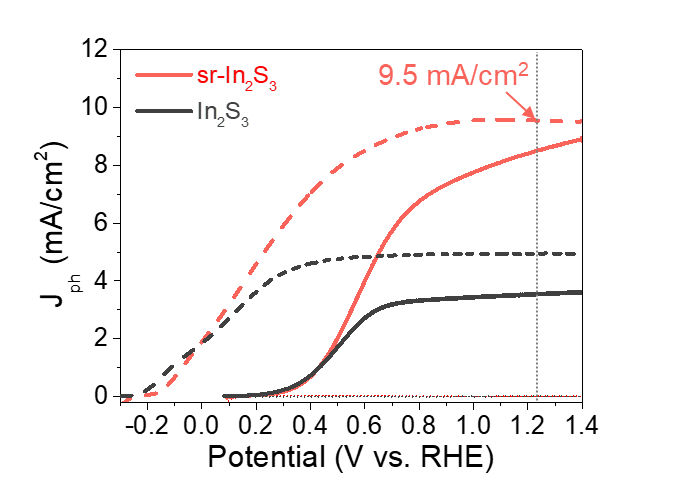


**Figure S32.** J-V curves in the presence of a hole scavenger (Na_2_SO_3_) under AM 1.5G (100 mW/cm^2^) illumination.


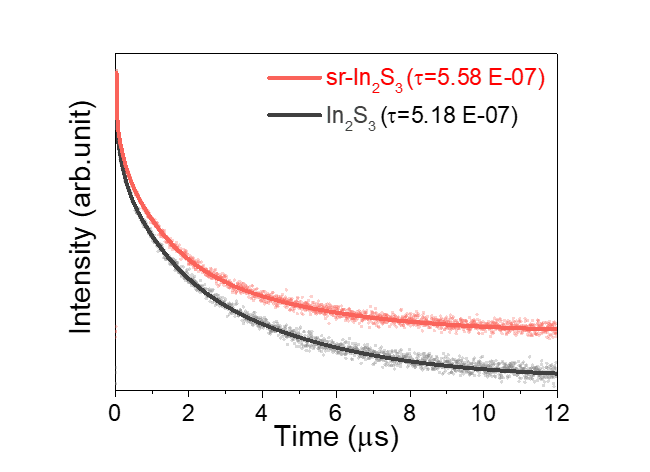


**Figure S33.** Time-resolved photoluminescence (TRPL) decay spectra of sr-In_2_S­_3_ and In_2_S_3_ photoanodes.


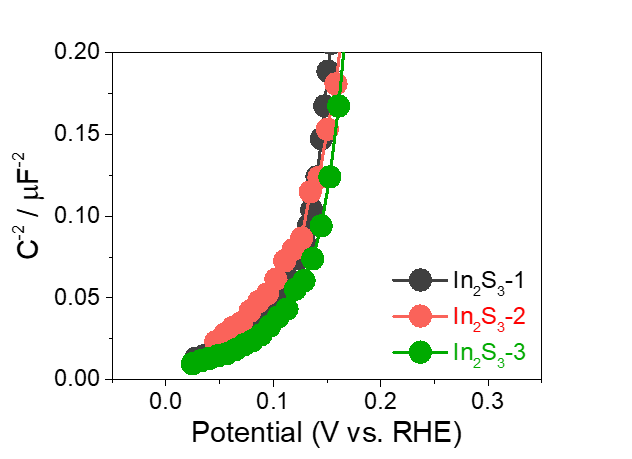


**Figure S34. Reconfirmation of Mott-Schottky plots on In_2_S_3_ photoanodes.** The results show identical slope and flat band potential of In_2_S_3_ photoanodes.


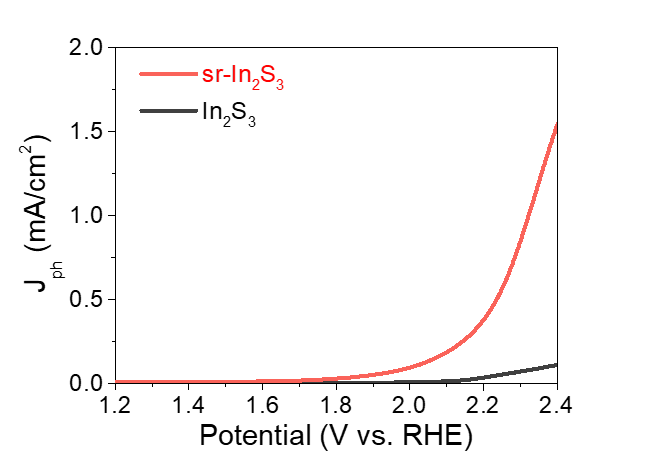


**Figure S35**. J-V curves of sr-In_2_S_3_ and In_2_S_3_ photoanodes in the dark condition with 0.5 M Na_2_SO_4_.


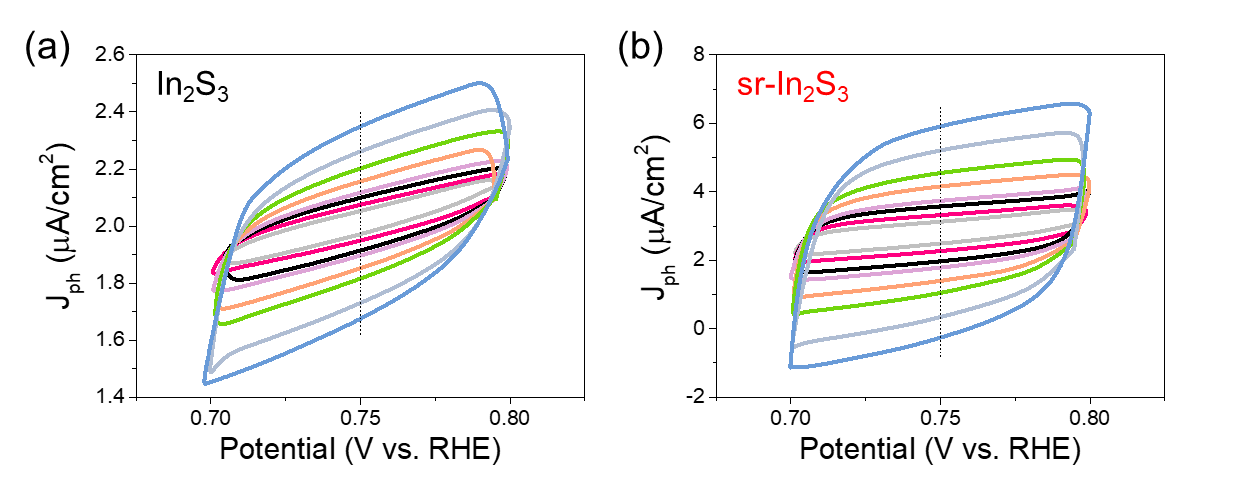


**Figure S36.** **Electrochemical active surface area (EASA) analysis.** Cyclic voltammetry (CV) curves of (a) In_2_S_3_ and (b) sr-In_2_S_3_ photoanodes measured at different scan rates (30-400 mV/s).


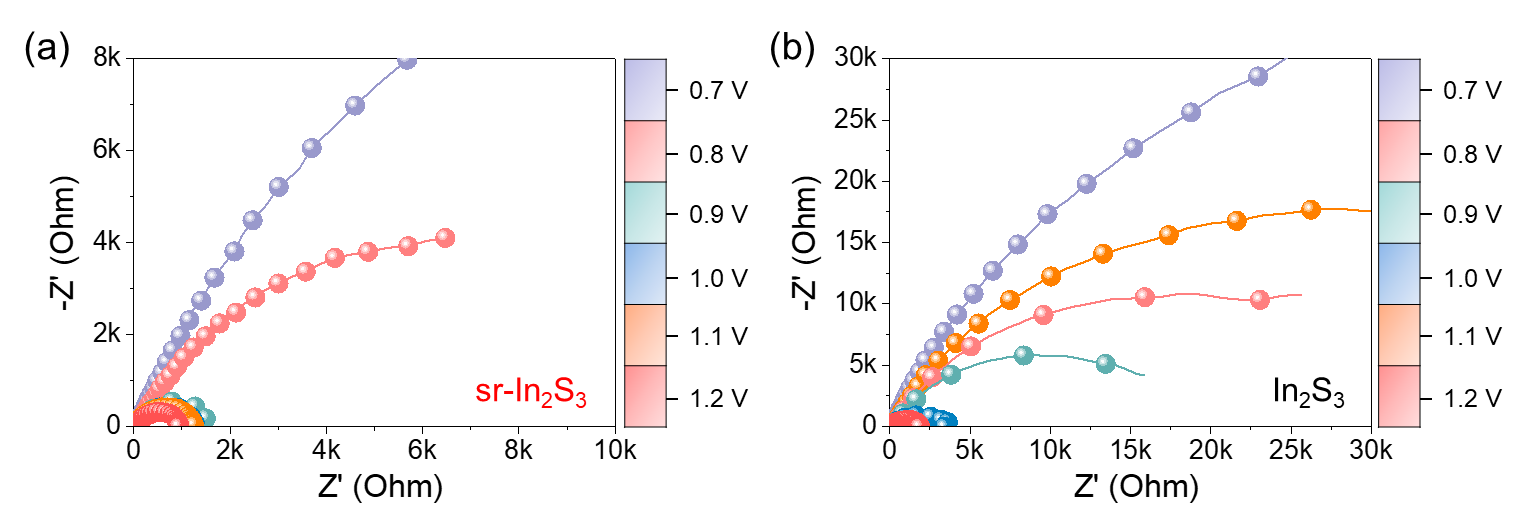


**Figure S37.** Potentiostatic electrochemical impedance spectroscopy (PEIS) analysis of (a) sr-In_2_S_3_ and (b) In_2_S_3_ photoanodes measured at different applied potentials (0.7-1.2 V).

**
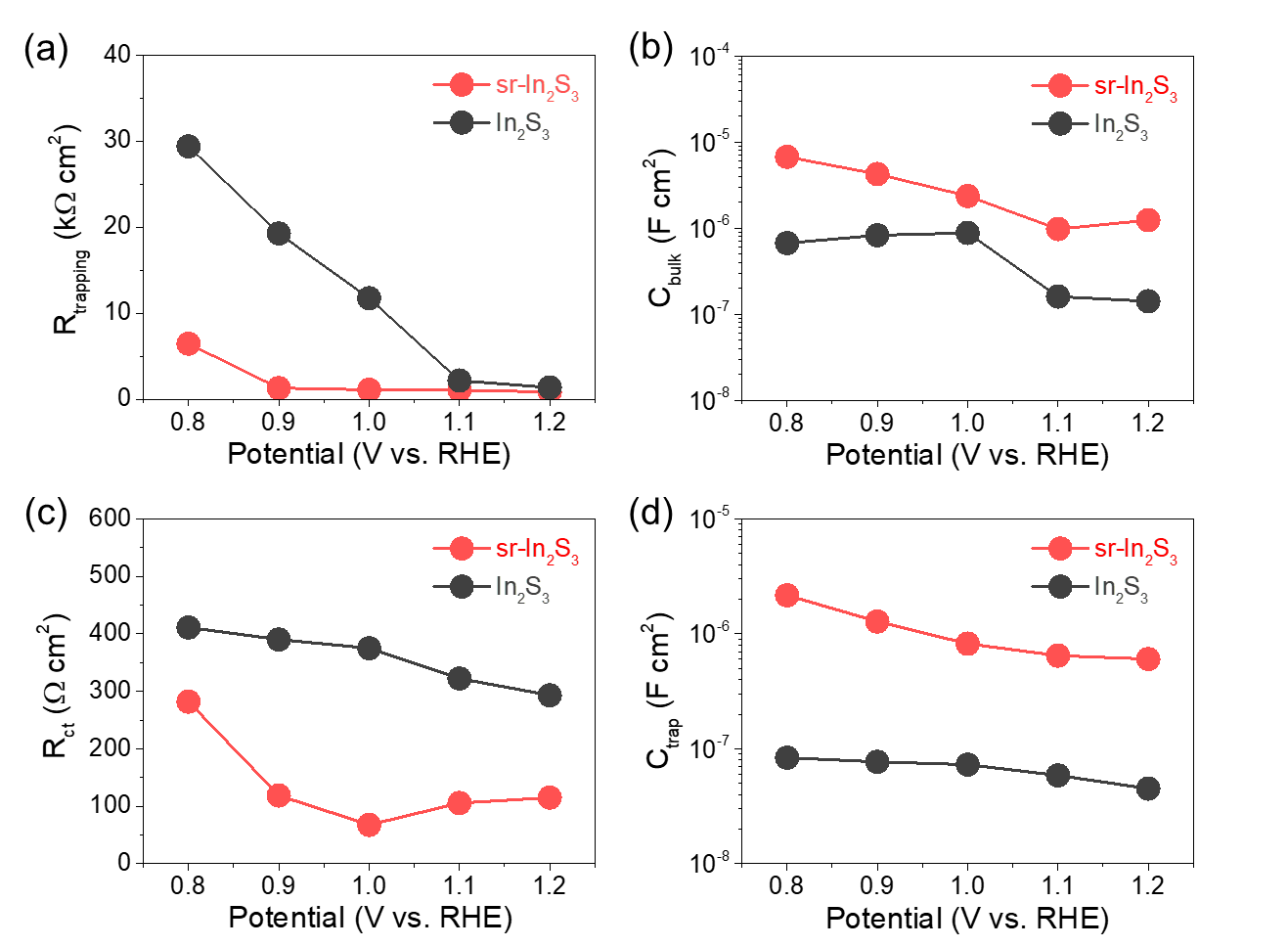
**

**Figure S38.** Photoelectrochemical impedance spectroscopy (PEIS) analysis of (a) R_trapping_ (resistance of hole trapping at the surface state), (b) C_bulk_ (capacitance of charge accumulation in the space charge layer), (c) R_ct_ (resistance between photoanode and electrolyte), and (d) C_trap_ (capacitance associated with charge accumulation on the surface states).

**References**

[1] H. Xu, H. Chen, S. Chen, K. Wang, X. Wang, *Int. J. Hydrogen Energy* **2021**, *46*, 32445.

[2] J. Hou, S. Cao, Y. Sun, Y. Wu, F. Liang, Z. Lin, L. Sun, *Adv. Energy Mater.* **2018**, *8*, 1701114.

[3] W. Xu, W. Tian, L. Meng, F. Cao, L. Li, *Adv. Mater. Interfaces* **2020**, *7*, 1901947.

[4] W. Xu, L. Meng, W. Tian, S. Li, F. Cao, L. Li, *Small* **2022**, *18*, 2105240.

[5] W. Xu, N. Fan, S. Xu, L. Meng, B. Xu, M. Zhou, W. Tian, L. Li, *Nanoscale* **2022**, *14*, 14520.

[6] H. Wang, Y. Xia, H. Li, X. Wang, Y. Yu, X. Jiao, D. Chen, *Nat. Commun.* **2020**, *11*, 3078.

[7] H. Wang, Y. Xia, N. Wen, Z. Shu, X. Jiao, D. Chen, *Appl. Catal. B Environ.* **2022**, *300*, 120717.

[8] L. Meng, S. Wang, F. Cao, W. Tian, R. Long, L. Li, *Angew. Chemie* **2019**, *131*, 6833.

[9] Y. Fu, F. Cao, F. Wu, Z. Diao, J. Chen, S. Shen, L. Li, *Adv. Funct. Mater.* **2018**, *28*, 1706785.

[10] L. Meng, C. Cheng, R. Long, W. Xu, S. Li, W. Tian, L. Li, *Sci. Bull.* **2022**, *67*, 1562.

[11] L. Meng, X. Zhou, S. Wang, Y. Zhou, W. Tian, P. Kidkhunthod, S. Tunmee, Y. Tang, R. Long, Y. Xin, *Angew. Chemie Int. Ed.* **2019**, *58*, 16668.

[12] Y. Wu, X. Liu, H. Zhang, J. Li, M. Zhou, L. Li, Y. Wang, *Angew. Chemie* **2021**, *133*, 3529.

[13] W. Tian, C. Chen, L. Meng, W. Xu, F. Cao, L. Li, *Adv. Energy Mater.* **2020**, *10*, 1903951.

[14] Y. Gao, S. Zhang, Y. Wu, Y. Tian, H. Fu, S. Zhan, *J. Catal.* **2019**, *375*, 389.

[15] L. Meng, M. Wang, H. Sun, W. Tian, C. Xiao, S. Wu, F. Cao, L. Li, *Adv. Mater.* **2020**, *32*, 2002893.

[16] H. Li, C. Chen, X. Huang, Y. Leng, M. Hou, X. Xiao, J. Bao, J. You, W. Zhang, Y. Wang, *J. Power Sources* **2014**, *247*, 915.

[17] B. Fan, Z. Chen, Q. Liu, Z. Zhang, X. Fang, *Appl. Surf. Sci.* **2016**, *370*, 252.

[18] G. Lv, L. Long, X. Wu, Y. Qian, G. Zhou, F. Pan, Z. Li, D. Wang, *Appl. Surf. Sci.* **2023**, *609*, 155335.

[19] S. Li, L. Meng, W. Tian, L. Li, *Adv. Energy Mater.* **2022**, *12*, 2200629.

[20] L. Meng, J. He, X. Zhou, K. Deng, W. Xu, P. Kidkhunthod, R. Long, Y. Tang, L. Li, *Nat. Commun.* **2021**, *12*, 5247.

[21] Y. Zhang, B. Zhou, Z. Wei, W. Zhou, D. Wang, J. Tian, T. Wang, S. Zhao, J. Liu, L. Tao, *Adv. Mater.* **2021**, *33*, 2104791.
